# Achieving high hybridization density at DNA biosensor surfaces using branched spacer and click chemistry†

**DOI:** 10.1039/d3ra04928k

**Published:** 2023-11-21

**Authors:** Alireza Kavand, Perrine Robin, Lucas Mayoraz, Mounir Mensi, Sandrine Gerber-Lemaire

**Affiliations:** a Group for Functionalized Biomaterials, Institute of Chemical Sciences and Engineering, Ecole Polytechnique Fédérale de Lausanne CH-1015 Lausanne Switzerland sandrine.gerber@epfl.ch; b ISIC-XRDSAP, EPFL Valais-Wallis Rue de l'Industrie 17 CH-1951 Sion Switzerland

## Abstract

The COVID-19 pandemic has highlighted the necessity to develop fast, highly sensitive and selective virus detection methods. Surface-based DNA-biosensors are interesting candidates for this purpose. Functionalization of solid substrates with DNA must be precisely controlled to achieve the required accuracy and sensitivity. In particular, achieving high hybridization density at the sensing surface is a prerequisite to reach a low limit of detection. We herein describe a strategy based on peptides as anchoring units to immobilize DNA probes at the surface of borosilicate slides. While the coating pathway involves copper-catalyzed click chemistry, a copper-free variation is also reported. The resulting biochips display a high hybridization density (2.9 pmol per cm^2^) with their targeted gene sequences.

## Abstract 2

Surface functionalization of glass involving peptide-based branched spacer and click chemistry allows for efficient covalent conjugation of single-strand DNA to achieve high hybridization density tailored for biosensing applications.

## Graphical abstract

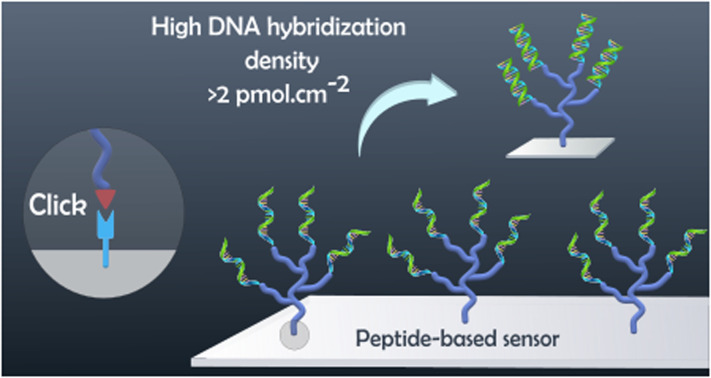

## Introduction

The development of DNA biosensors accelerated in the past decade, especially for the purpose of medical diagnosis, cancer research and gene expression analysis.^1^ The recent COVID-19 pandemic stressed the necessity to develop sensitive and reliable virus detection techniques. Surface-based DNA biosensors provide numerous benefits over other types, such as high sensitivity and affordability.^2^ They can also be implemented in microfluidic systems for automated detection.^3^ These sensors rely on the immobilization of single-stranded DNA (ssDNA) probes on solid substrates that are capable of hybridization with their complementary DNA or RNA targeted sequence.

Among others, the probe density and hybridization efficiency of ssDNA probes immobilized on the surface are crucial parameters for the performance of the biosensing device.^3,4^ While the probe density corresponds to the number of probes attached on a certain area, the hybridization efficiency refers to the number of probes accessible to hybridization with their complementary target on a certain area. To increase the hybridization efficiency, a maximum of available binding sites should be displayed to provide a large number of anchoring sites for the targeted molecule, hence increasing the sensitivity of the sensor. However, the lateral spacing between the probes should be controlled to avoid crowding effect.^3–6^

Surface attachment of DNA probes is usually carried out *via* physical adsorption, chemical conjugation, or (strept-)avidin–biotin interaction.^4^ One of the most common methods for covalent immobilization of ssDNA is the formation of self-assembled monolayers of organosilanes which can introduce different reactive groups on the surface, *e.g.* amino, aldehyde, carboxylic acid, epoxy, and isothiocyanate on a variety of surfaces.^7–9^ Silanization emerged as a suitable strategy for the chemical conjugation of DNA probes to sensing surfaces due to the large variety of commercial silane derivatives, as well as the straightforwardness and affordability of the procedure.

One method to increase the hybridization efficiency of DNA biosensors is to introduce a spacer that lifts the probes off the surface and ensures distancing between the immobilized strands.^10,11^ Furthermore, spacer molecules could promote the orientation of the probe for more efficient hybridization,^12^ and improve the resistance to non-specific surface adsorption.^13,14^ Many reports disclosed the introduction of either vertical or lateral spacing units on the surface, for instance, 6-mercapto-1-hexanol and poly(ethylene glycol),^15,16^ poly-thymine (polyTm) sequences of different lengths,^17^ short poly(ethylene)glycols, mercapto-alkyl spacers,^18^ poly(dT) spacers,^19^ poly-guanine (poly(dG)),^12,20^ adenine oligonucleotide and thymine.^21^

For the engineering of biosensors, peptides stand as an important class of materials, allowing a large variety of applications.^21–23^ In this context, peptides can not only serve as bioreceptors, but also as anchoring units. Indeed, several studies highlighted the use of peptides to conjugate immobilizing recognition elements on sensing surfaces. For example, a zwitterionic peptide was reported to immobilize ssDNA probe on poly(3,4-ethylenedioxythiophene) for the detection of breast cancer marker.^24^ In another study, aptamers were immobilized to a new sequence of peptides onto polyaniline substrate for detecting cancer cells.^25^ Compared to other spacers, peptides have the advantage to be easily tunable in terms of length and functional groups through the iterative addition of amino acid-based building blocks on a solid support.

The present study was devoted to the development of branched linkers for the immobilization of ssDNA probes on solid support, in order to achieve a high hybridization density with their targeted analytes. Peptides were selected as anchoring molecules to serve both high-density probe immobilization and lateral spacing purposes. Regarding the solid substrate, glass was chosen for its affordability, stability and suitability for fluorescence detection, commonly used as transducing method for DNA biosensors.^3^

Solid phase peptide synthesis (SPPS) was applied to the preparation of two peptide-derived spacers based on glutamic acid with N-terminus functional azide or alkyne groups. In view of the versatility of click reactions for biomaterials functionalization,^26,27^ we focused on copper-catalyzed and strain-promoted azide to alkyne [3 + 2] cycloaddition (CuAAC and SPAAC) reactions for peptide conjugation to the sensing slides, followed by covalent immobilization of ssDNA probe to the peptides displayed at the surface. In this study, we selected a previously reported probe sequence that specifically targets SARS-CoV-2 ^28^ as model for ssDNA sequence to be immobilized. Noteworthy, the method used for conjugating DNA on the surface is independent of the nucleic acid sequence and could be thus implemented for other DNA probe sequences.

Each step of the functionalization pathway was monitored by high resolution X-ray photoelectron spectroscopy (XPS) to identify and characterize the chemical composition of the added layers. Then, we evaluated and compared the achieved surface hybridization density to previous studies, as this parameter directly measures the capacity of a sensing surface to capture its targeted biomolecules.

## Results and discussion

**Scheme 1:**
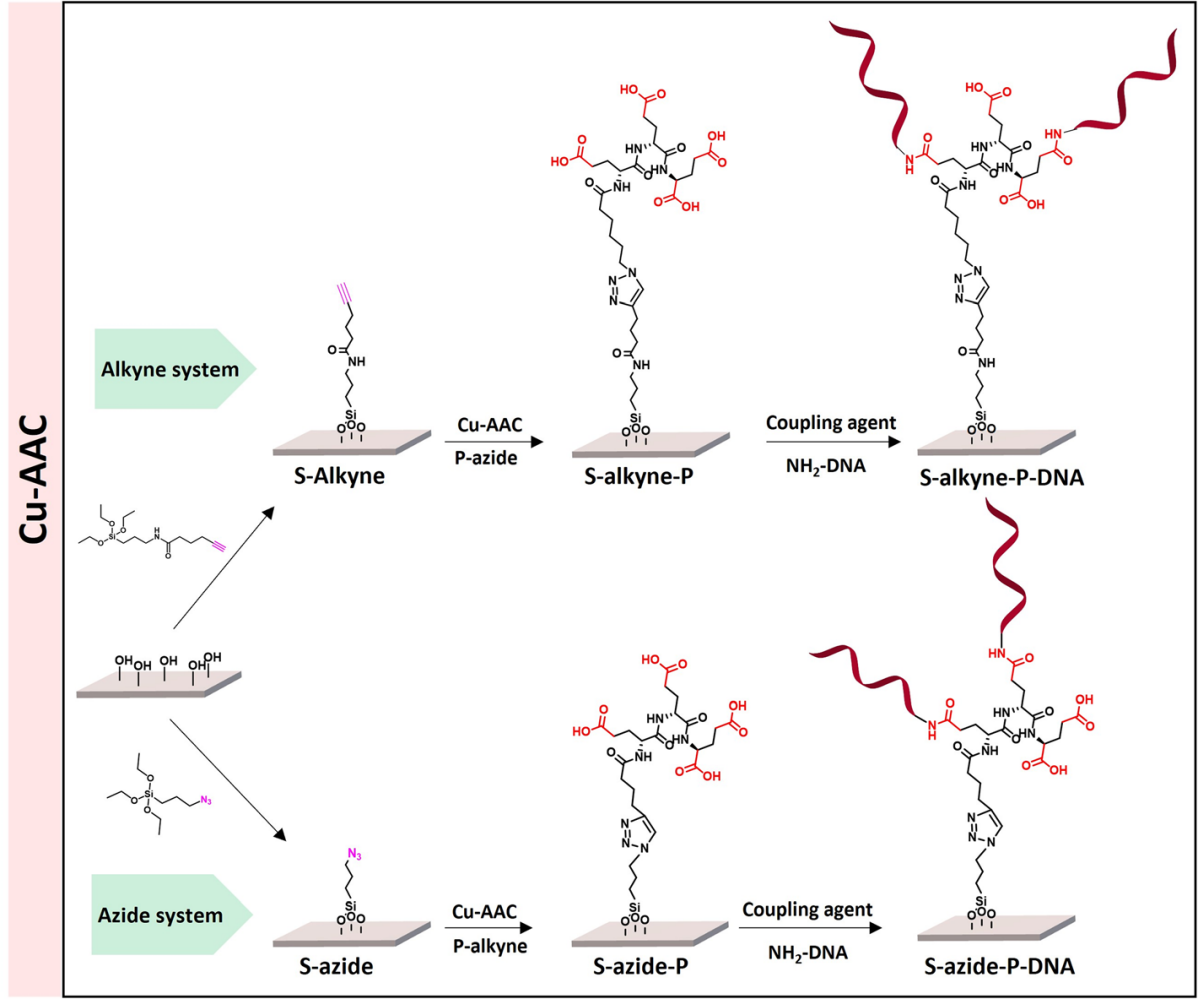
Overview of the different peptide-based systems developed for S-OH slides functionalization.

The sequential functionalization of borosilicate slides (S-OH) started by silanization to introduce surface reactive azide or alkyne functionalities, followed by CuAAC for conjugation to cross-reactive branched spacer based on peptides containing four carboxylic groups, to deliver peptide-conjugated surfaces S-alkyne-P and S-azide-P (Scheme 1). The final step made use of amide-bond formation for immobilization of the DNA probe sequence.

### Peptide synthesis

**Scheme 2:**
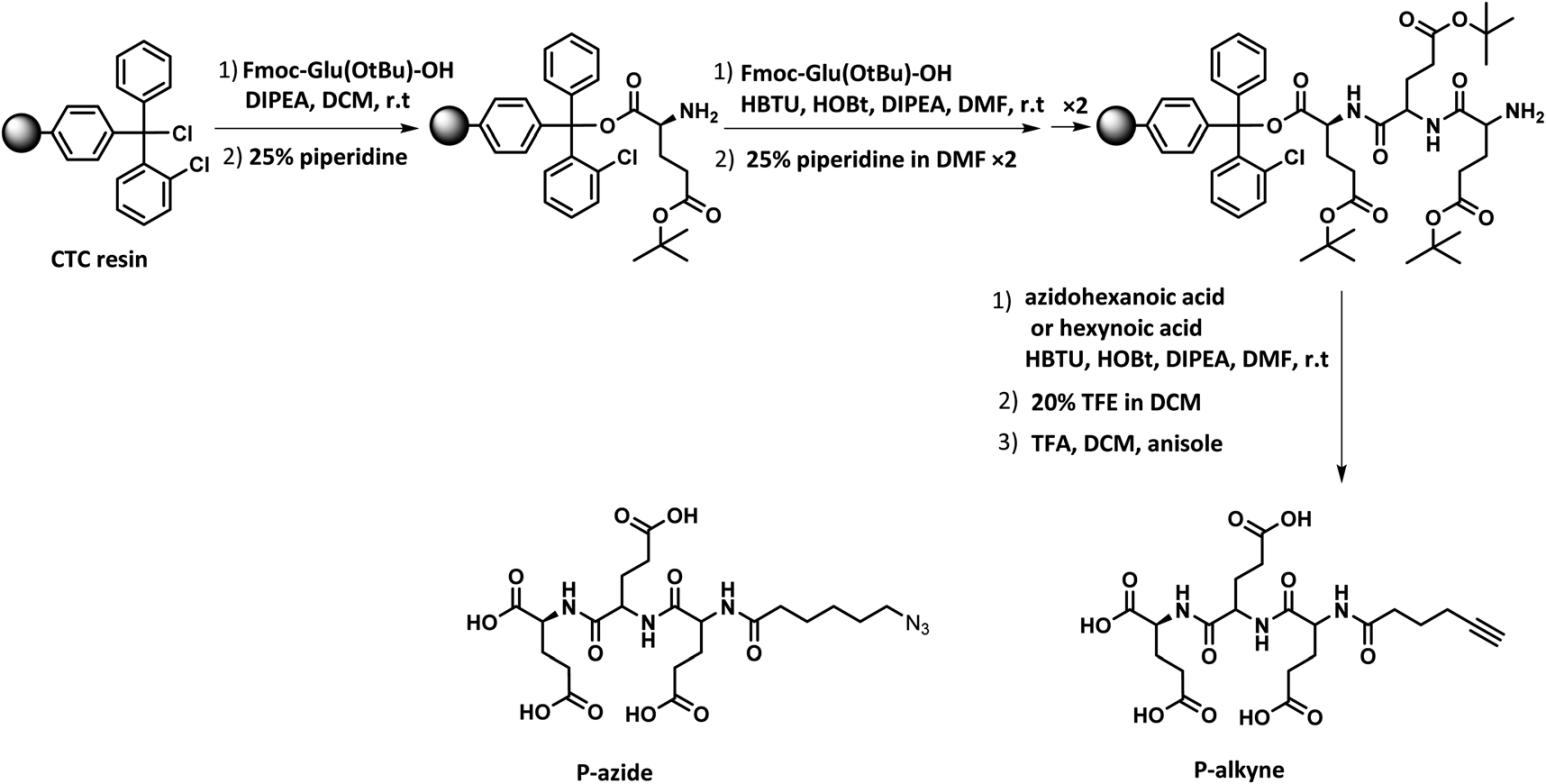
Solid phase peptide synthesis (SPPS) of P-azide and P-alkyne.

The peptides were prepared by SPPS from a 2-chlorotrityl chloride resin adding iteratively Fmoc-Glu(OtBu)-OH and using 2-(1*H*-benzotriazol-1-yl)-1,1,3,3-tetramethyluronium hexafluorophosphate (HBTU) as coupling agent (Scheme 2). After three iterations, the sequence was end-capped with either azidohexanoic acid or hexynoic acid to equip the peptides with azide and alkyne functionalities. Cleavage from the resin was achieved in the presence of trifluoroethanol (TFE), followed by acidic removal of the protecting groups to afford P-alkyne and P-azide in high yield. The structure and purity of the peptides were confirmed by NMR analysis and HRMS (ESI, Fig. S1–S8†).

### Preparation of peptide-conjugated surfaces

The introduction of surface reactive functionalities was achieved by silanization in the presence of derivatives of (3-aminopropyl)triethoxysilane (APTES) holding terminal alkyne or azide functionalities (for the synthesis of silanization reagents APTES-N_3_ and APTES-Alkyne, see ESI, Section 2.2†).

S-OH slides were activated by plasma treatment (17 min), followed by immersion in a solution of silanization reagents in dry toluene, under inert atmosphere for 48 h.

**Fig. 1:**
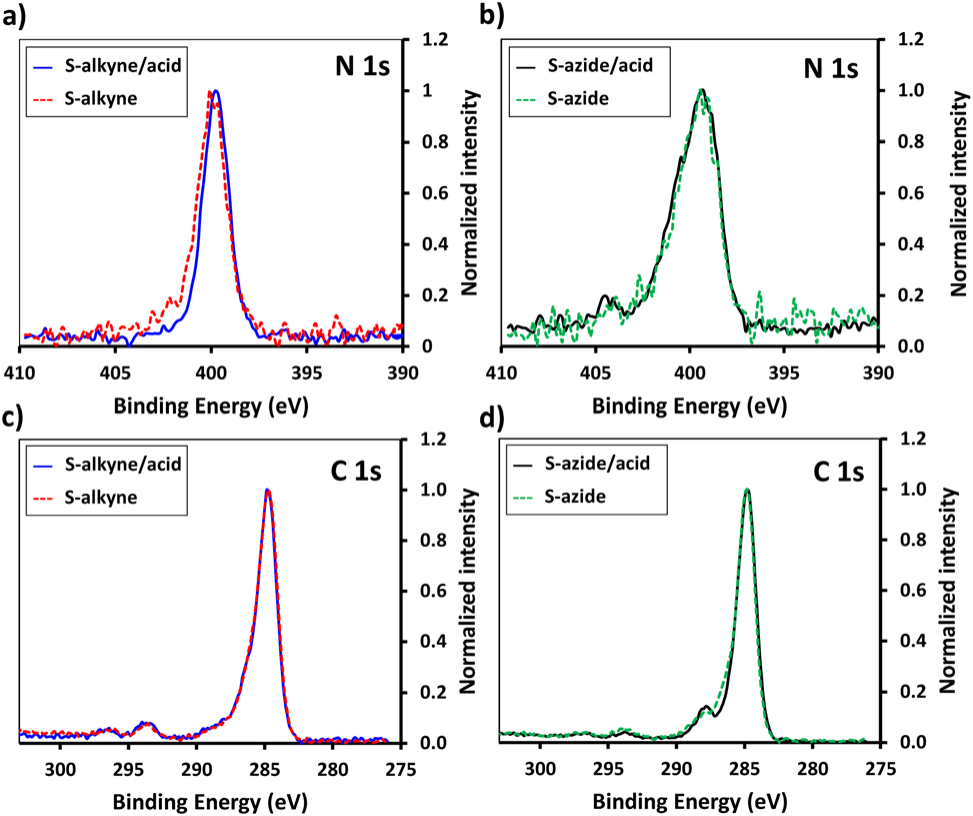
High resolution XPS spectra of N 1s scans of S-alkyne (a) and S-azide (b) slides produced with and without valeric acid addition. C 1s XPS high-resolution spectra of S-alkyne (c) and S-azide (d) slides produced with and without valeric acid addition.

In order to enhance the hydrolysis rate during the silanization process, the use of valeric acid as additive was investigated. As reported in the literature, post-treatment of the modified slides by heating at 80 °C for 1.5 h was applied to promote condensation and siloxane bond formation.^29^ The silanization step was verified by XPS, tracking the characteristic peaks of the silane derivatives introduced on the surface (Fig. 1). The S-alkyne slide surfaces displayed a N 1s peak as single component at 400.0 eV and a C 1s peak at 284.8 eV with a shoulder on the high energy side (Fig. 1a and c) corresponding to C–N at 285.9 eV and amide bond (O=C–N) at 287.9 eV, respectively. Due to the presence of both alkylamino- and azido-nitrogen atoms,^25^ the XPS survey spectra of S-azide slides revealed N 1s peaks at 399.3 and 404.4 eV (Fig. 1b). Given that the silicon peaks mainly originate from the SiO_2_ substrate, implying a consistent Si concentration, we employed the C/Si and N/Si ratios as an additional parameter to assess the quantity of deposited APTES-alkyne and APTES-N_3_ across all layers. The ratio of C/Si and N/Si was increased when the silanization step was conducted in the presence of the valeric acid additive, pointing toward the rate-enhancing effect of the acid for surface siloxane condensation (for comparison of full XPS survey spectra and percentage of elemental composition, see ESI Fig. S25 and Table S1,† respectively).^30^

Further characterization of silanized surfaces was performed by quantification of reactive alkyne and azide functionalities, using cleavable and clickable fluorescent labels, as suggested by Miyahara *et al.*^31^ The procedure is illustrated in ESI, Fig. S24.† The synthesis of the labelling reagents are detailed in ESI (Section 2.3).†S-Alkyne slides prepared in the presence of valeric acid were characterized by an average quantity of reactive alkyne groups of 692 ± 86 pmol cm^−2^ (Table 1). Under such conditions, a closely packed layer of reactive functionalities was achieved, corresponding to a grafting density of 4.2 ± 0.5 molecules per nm^2^. The impact of the addition of valeric acid additive was less pronounced for the silanization with APTES-azide, which resulted in available reactive azido groups in 202 ± 14 (with acid) and 72 ± 53 (without acid) pmol cm^−2^ quantities.

**Table 1:** Quantification of surface reactive groups on S-alkyne and S-azide slides. Results are expressed as the mean ± SD of *n* independent experiments

Slides	Reactive groups [pmol cm^−2^]	Grafting density [molecules per nm^2^]
S-Alkyne^a^	20 ± 28 (*n* = 2)	0.19 ± 0.16
S-Alkyne^b^	692 ± 86 (*n* = 5)	4.2 ± 0.5
S-Azide^a^	72 ± 53 (*n* = 4)	0.4 ± 0.3
S-Azide^b^	202 ± 14 (*n* = 2)	1.2 ± 0.1

a Silanization was performed in the presence of APTES-alkyne or APTES-azide (4.8 mM), in dry toluene.

b Silanization was performed in the presence of APTES-alkyne or APTES-azide (4.8 mM) and valeric acid (3.7 mM), in dry toluene.

For further functionalization studies, the acid-promoted silanization protocol was systematically applied in order to ensure high surface loading of the designed peptides. In addition, these conditions resulted in higher batch-to-batch reproducibility.

**Fig. 2:**
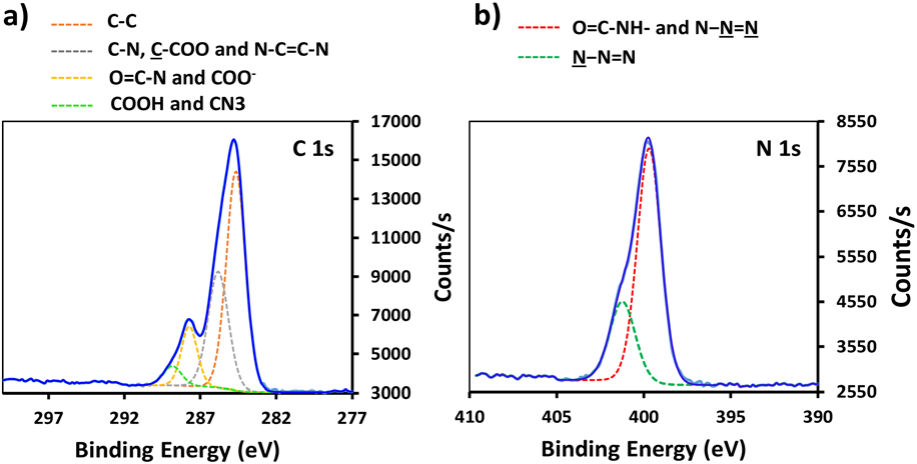
High resolution XPS data of peptide-functionalized slide: (a) C 1s XPS spectrum of S-alkyne-P and (b) N 1s spectrum of S-alkyne-P.

Conjugation of P-alkyne and P-azide peptides to cross-reactive silanized slides was achieved through CuAAC reaction, in the presence of CuSO_4_ and sodium ascorbate. Several parameters, such as reaction time, temperature, solvent composition, peptide concentration and addition of tris(3-hydroxypropyltriazolylmethyl)amine (THPTA) as Cu(i) stabilizing ligand,^32^ were varied (Table 2). The effect of the different conditions was assessed by quantification of the hybridization density following the final step of DNA probe immobilization, and will be discussed in the next section. S-Alkyne slides were preferentially investigated due to their higher amount of surface available reactive groups. Following CuAAC reaction with P-azide, the slides were thoroughly washed with Tween-20 (0.1%) or Cyclam solution (2 mg mL^−1^) to remove residual copper species from the functionalized surface, and further analyzed by XPS measurements. The C 1s and N 1s binding energy regions gave evidence for the peptide attachment to the surface. Fitting of the C 1s signal confirmed the presence of different chemical environments for the carbon atoms of the peptide backbone and triazole ring (Fig. 2). Similarly, fitting of the N 1s signal revealed characteristic peaks for both the peptide chain and the triazole ring. The C/N elemental ratio was estimated to be 4.8, which is close to the stoichiometric value of C/N = 4.3 (percentage of elemental composition of peptide-conjugated slides is provided in ESI, Table S2†). The slight excess observed in carbon can be attributed to a minor amount of atmospheric contamination. We also verified the absence of peaks in the Cu 2p region of the XPS spectrum, following the washing procedure with either Tween 20 or Cyclam solution (ESI, Fig. S26†).

**Table 2:** Screening of experimental conditions for peptide conjugation through CuAAC click reactions. The performance of the coating layer was assessed by quantification of the hybridization density of immobilized DNA probe at the end of the functionalization sequence

S-x	Entry	*t* (h)	*T* (°C)	THPTA^a^ (mM)	Peptide (mM)	Solvent^b^	Hybridization density (pmol cm^−2^)
S-Alkyne	1	4	25	0	1.8	ddH_2_O	0.76
	2	4	25	0.7	1.8	ddH_2_O	2.3 ± 0.4 (*n* = 3)^c^
	3	24	25	0.7	1.8	ddH_2_O	2.2
	4	4	37	0.7	1.8	ddH_2_O	1.2
	5	20	37	0.7	1.8	ddH_2_O	1.3
	6	4	37	0.7	3.6	ddH_2_O	2.1
	7	4	25	0.7	3.6	ddH_2_O	2.3
	8	4	25	1.4	3.6	ddH_2_O	2.9
	9	4	25	0.7	3.6	ddH_2_O/MeOH	2.9 ± 0.8 (*n* = 3)^c^
	10	24	25	0.7	3.6	ddH_2_O/MeOH	2.9
S-Azide	11	4	25	0	3.6	ddH_2_O	0.8
	12	4	25	0.7	3.6	ddH_2_O	1.5
	13	4	25	0.7	3.6	ddH_2_O/MeOH	1.4

a THPTA: tris((1-hydroxy-propyl-1*H*-1,2,3-triazol-4-yl)methyl)amine.

b ddH_2_O: double-distilled water.

c Reproducibility of the conditions was assessed on *n* = 3 independent experiments.

The click reaction protocol was also applied to S-azide slides, in the presence of P-alkyne peptide, giving rise to conjugated slides presenting similar patterns in XPS measurements (see ESI, Fig. S27†).

**Fig. 3:**
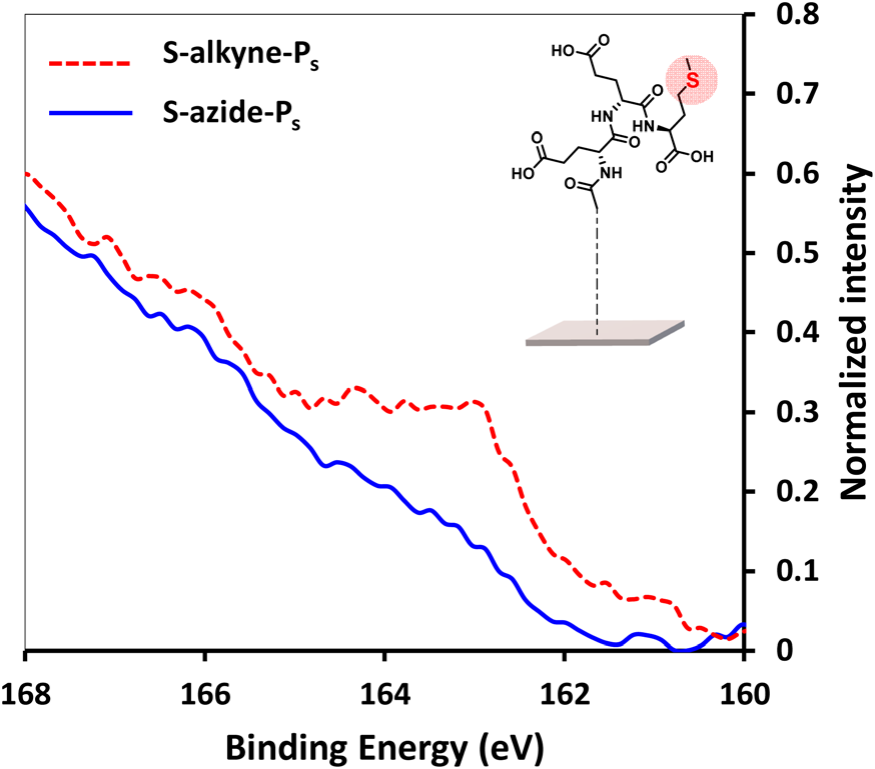
High resolution XPS spectra of S 2p scans of S-alkyne-P_s_ and S-azide-P_s_. Reaction conditions: 3.6 mM of peptide, CuSO_4_, THPTA, H_2_O/MeOH 1 : 1, 4 h, 25 °C.

In order to facilitate the monitoring of surface conjugation to the designed peptides, analogue sequences incorporating a methionine residue were synthesized for further detection of the S 2p signal in XPS spectra (synthesis of P_s_-azide and P_s_-alkyne peptides is detailed in ESI, Section 2.1†). After click reaction of S-alkyne slides with P_s_-azide peptide, XPS analysis of the resulting surfaces showed the appearance of a S 2p peak (Fig. 3), along with an increase of the intensity of both C 1s and N 1s peaks (ESI, Fig. S28†). These results are in agreement with the successful conjugation of the peptide to the silanized surface.

### Immobilization of ssDNA probes on peptide-functionalized slides

Following peptide anchoring *via* click chemistry, the covalent attachment of ssDNA probe targeting the SARS-CoV-2 viral genome was investigated. According to the procedure developed in a previous study,^28^ the combination of *N*-(3-dimethylaminopropyl)-*N*-ethylcarbodiimide and 1-hydroxybenzotriazole hydrate (EDC/HOBt) was used for activation of peptide carboxylic acids, followed by coupling to amino-modified DNA probe (NH_2_-DNA, sequence is given in Materials and methods).

**Fig. 4:**
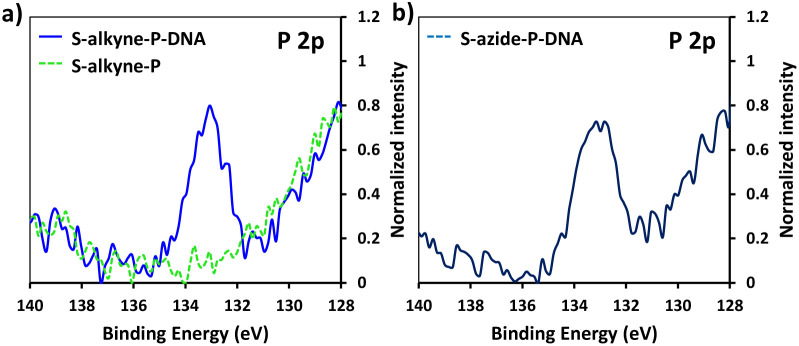
High resolution XPS spectra of P 2p scans of S-alkyne-P and S-alkyne-P-DNA (a) and S-azide-P-DNA (b).

XPS analysis of the resulting S-alkyne-P-DNA and S-azide-P-DNA slides was used to confirm DNA immobilization by monitoring the appearance of a P 2p signal at 133.2 eV (Fig. 4, high resolution XPS spectra of C 1s and N 1s scans of S-alkyne-P-DNA and S-azide-P-DNA and elemental composition of the analyzed slides are provided in ESI, Fig. S29 and Table S3,† respectively). It is worth mentioning that the P 2s peak of DNA molecules appeared at 190.5 eV (ESI, Fig. S29†). This energy value is relatively close to the binding energy of B 1s at 193.0 eV, which is characteristic of borosilicate substrates, which prompted us to monitor the P 2p signal for probing DNA conjugation.

The hybridization density of NH_2_-DNA probes immobilized at the surface of the glass slides was evaluated following the procedure described in our previous study.^28^ Notably, the hybridization density refers to the concentration of DNA molecules that have successfully bound to their complementary DNA strands on the surface. The hybridization density values are depicted in Table 2. Variation on the reaction parameters of the click reaction used in the peptide conjugation step had a strong impact on the final hybridization density of the immobilized DNA probe. Starting from S-alkyne slides, the addition of the THPTA coordinating ligand led to a significant increase of the hybridization density from 0.76 to 2.3 pmol cm^−2^ (entries 1 and 2). Prolonged reaction time (20 h *vs.* 4 h, entries 2 and 3) and higher temperature (37 °C *vs.* 25 °C, entries 2 and 4) resulted in a decrease of the hybridization density, that may be due to degradation of the surface. Also, increasing the concentration of both the peptide and the coordinating ligand afforded a higher density of immobilized DNA available for hybridization (entry 8). Finally, moving from pure ddH_2_O to a mixture of ddH_2_O/MeOH did not have a significant impact on the performance of the immobilization step. Overall, the higher hybridization density on S-Alkyne-P slides was obtained by performing the peptide conjugation for 4 h at 25 °C, in a mixture of ddH_2_O/MeOH and in the presence of THPTA ligand, using a peptide concentration of 3.6 mM (entry 9). Under those conditions, the functionalization pathway led to a hybridization density of 2.9 ± 0.8 pmol cm^−2^ (*n* = 3 independent experiments). Such value is significantly higher than the hybridization density recently reported for DNA immobilization on three-dimensional surface structures (*i.e.* hybridization density = 0.43 pmol cm^−2^).^33^

In agreement with the lower amount of surface reactive groups on S-azide slides, the hybridization density of S-azide-P-DNA surfaces culminated at 1.5 pmol cm^−2^ (entry 12).

The stability of the S-alkyne-P-DNA slides was assessed by performing the quantification of immobilized DNA available for hybridization after storage at 4 °C in MiliQ for 4 weeks (ESI, Table S4†). No significant variation of the hybridization density was observed, indicating the stability of the functional layer for at least one month.

### Copper-free conjugation strategy for the engineering of DNA biosensors

The implementation of CuAAC for the conjugation of the peptides to silanized S-alkyne surfaces led to high DNA hybridization density. XPS measurements on the resulting S-alkyne-P-DNA slides did not reveal the presence of copper, pointing toward the effectiveness of the washing procedure with Tween 20 (ESI, Fig. S26†). However, we believe that a metal-free procedure would reduce the needs for multiple washing cycles and ensure the absence of residual copper traces, which might be deleterious for certain types of detection such as electrochemical detection or surface plasmon resonance.

**Scheme 3:**
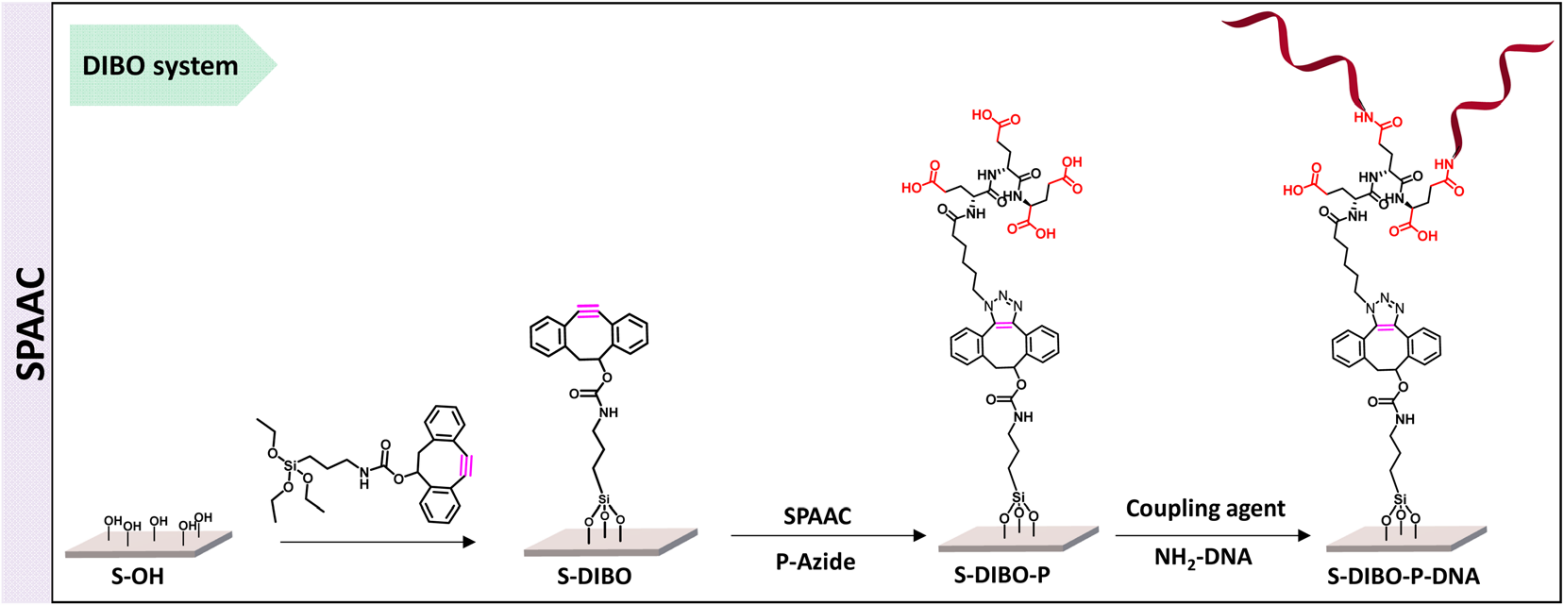
Schematic illustration of DNA immobilization strategy using copper-free click chemistry.

**Fig. 5:**
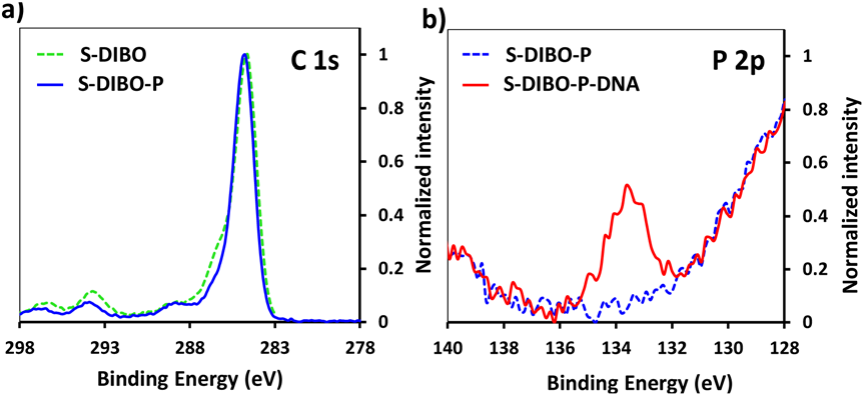
(a) XPS spectrum of C 1s signal of borosilicate substrate before and after silanization with APTES-DIBO. (b) XPS spectrum of P 2p signal of S-DIBO-P and S-DIBO-P-DNA slides.

Therefore, a similar functionalization procedure (illustrated in Scheme 3) was developed based on SPAAC for peptide conjugation to silanized surfaces. The silanization reagent APTES-DIBO (synthesis detailed in ESI, Section 2.2†) was reacted with S-OH slides, in the presence of valeric acid, in dry toluene. The silanization was verified *via* XPS (Fig. 5a) and the amount of surface available DIBO functionalities was evaluated at 66 ± 19 pmol cm^−2^ (*n* = 3, independent experiments) *via* the quantification method with the N_3_-cleavable-FITC labelling reagent (ESI, Section 2.3†).

The resulting S-DIBO slides were then immersed in a solution of P-azide for conjugation through SPAAC reaction. Attachment of the peptide was verified by XPS analysis (Fig. 5a), monitoring the intensity of the C 1s signal at the different stages of the functionalization protocol. Final immobilization of the DNA probe was performed according to the protocol described above. Appearance of a P 2p signal in the XPS spectrum of S-DIBO-P-DNA slides gave evidence for the successful DNA immobilization (Fig. 5b).

The influence of the reaction time and temperature on the outcome of the SPAAC reaction was studied (Table 3) and monitored by quantification of the hybridization density at the end of the functionalization sequence.

**Table 3:** Hybridization density measured at the surface of S-DIBO-P-DNA slides. When repeated, the results are presented as mean values ± SD (*n* independent experiments). All reactions were performed in MeOH with a P-azide concentration of 3.6 mM

Entry	*t* (h)	*T* (°C)	Hybridization density (pmol cm^−2^)
1	4	25	1.08 ± 0.16 (*n* = 2)
2	20	25	1.65
3	4	37	2.3 ± 0.2 (*n* = 4)
4	4	50	0.47

Prolonged reaction time (20 *vs.* 4 h) resulted in a moderate increase of the hybridization density (entries 1 and 2). Rising the temperature from 25 to 37 °C had a beneficial effect on the peptide conjugation step (entries 1 and 3), resulting in a final hybridization density of 2.3 ± 0.2 pmol cm^−2^ (*n* = 4 independent experiments), which is similar to the values obtained *via* CuAAC reaction. However, further increase of the temperature to 50 °C led to a drastic decrease of the hybridization density, probably due to surface degradation. Overall, we recommend to select the conditions of entry 3 for the peptide conjugation step, allowing fast reaction time. It is to be noted that as a negative control, the hybridization density was measured on S-DIBO-P slides, incubated at 37 °C for 4 h, in the absence of DNA probe (0.33 ± 0.38 pmol cm^−2^, *n* = 4 independent experiments).

Finally, the stability of S-DIBO-P-DNA slides (SPAAC performed at 37 °C for 4 h) was evaluated as previously described (ESI, Table S4†), showing the integrity of the functionalized surface upon storage for 4 weeks at 4 °C in MilliQ water.

S-DIBO-P-DNA slides displayed similar performance towards hybridization than S-alkyne-P-DNA slides, highlighting the potential of the SPAAC conjugation route for the engineering of DNA biosensors. This immobilization strategy could be therefore suitable for detection techniques that would require metal-free sensors such as electrochemical biodetection.

In Table 4, the amount of hybridization density reported in previous studies is detailed. While the hybridization densities are generally around 0.25 to 0.8 pmol cm^−2^, a study from Miyahara *et al.* achieved from 1 to 5.4 pmol cm^−2^, depending on the concentration of DNA probes used for the functionalisation (1 to 8 μM probe solutions, corresponding to 0.2 to 1.6 nmol probe spotted on the surface).^31^ In comparison, the strategy described in this paper is performed in solution, with 0.5 μM probe concentration only. Due to a fully covalent functionalization strategy, the methodology herein proposed does not require the DNA to be deposited as a droplet on the surface, but works by fully immersing the carboxylate-decorated substrate in a solution of amino-modified DNA probe. We therefore believe that this procedure may lead to more homogeneous coatings, and is more versatile as the nature of the silanization reagent and the topology of the peptide spacer can be easily modulated. It can be applied to a variety of substrate dimensions, and is easier to control for large production. Finally, covalent immobilization of all coating layers ensures long term stability of the resulting functionalized surfaces as demonstrated by their preserved integrity upon storage in MilliQ water at 4 °C, for 4 weeks.

**Table 4:** Comparison of the obtained hybridization efficiency of the surface described in this work to previous studies

Type of surface	Hybridization density (pmol cm^−2^)	Reference
Glass coated with poly (PEGDA-*co*-GMA)	0.25	Qi *et al.*^34^
Glass coated with divinyl sulfone as homobifunctional crosslinker	0.33^a^	Cheng *et al.*^5^
Glass coated with cyclic olefin copolymer	0.43	Qi *et al.*^33^
PMMA	0.75	Fixe *et al.*^35^
Silicon surface coated with biotin	0.9	Escorihuela *et al.*^36^
PMMA	5.4	Miyahara *et al.*^31^
Glass, S-alkyne-P-DNA	2.9 ± 0.8 (*n* = 3)	This work
Glass, S-DIBO-P-DNA	2.3 ± 0.2 (*n* = 4)	This work

a This value was calculated from the probe density (0.88 pmol cm^−2^), multiplied by the hybridization efficiency (38.2%), resulting in a hybridization density of 0.33 pmol cm^−2^.

In conclusion, the methodology herein disclosed leads to the engineering of functionalized glass surfaces with the potential to reach a high hybridization density, as compared to previously reported studies. Further work would be required to evaluate the performance of these surfaces for the detection of targeted nucleic acids.

## Materials and methods

### Synthesis procedures

#### Peptide synthesis

The P-azide and P-alkyne sequences were synthesized by solid phase peptide synthesis techniques as described in Scheme 2. A similar procedure was applied to the synthesis of P_s_-azide and P_s_-alkyne sequences. Details about the procedure and characterizations are available in ESI (Section 2.1, Fig. S1–S8†).

#### Analytical data for P-azide

^1^H NMR (400 MHz, MeOD): *δ* 4.38–4.21 (m, 3H), 3.20–3.17 (m, 2H), 2.43–2.26 (m, 6H), 2.25–1.96 (m, 5H), 1.84 (dddd, *J* = 14.1, 8.9, 7.0, 4.9 Hz, 3H), 1.77–1.44 (m, 4H), 1.41–1.24 (m, 2H). HRMS (ESI/QTOF) *m*/*z*: [M + H_−1_]^−^ calcd for C_21_H_31_N_6_O_11_^−^ 543.2056; found 543.2045.

#### Analytical data for P-alkyne

^1^H NMR (400 MHz, MeOD): *δ* 4.49–4.33 (m, 3H), 2.51–2.34 (m, 8H), 2.28–2.05 (m, 6H), 2.04–1.88 (m, 3H), 1.87–1.75 (m, 2H). HRMS (ESI/QTOF) *m*/*z*: [M + H_−1_]^−^ calcd for C_21_H_28_N_3_O_11_^−^ 498.1729; found 498.1742.

#### Cleavable fluorophore synthesis

The synthesis protocols of the cleavable fluorescent derivatives used for the quantification of surface reactive functionalities are available in ESI (Section 2.3).†

#### Analytical data for N_3_-cleavable-FITC

^1^H NMR (400 MHz, MeOD) *δ* 8.33–8.31 (m, 1H, C*H*-Ar), 7.90–7.87 (dd, 1H, C*H*-Ar), 7.23–7.18 (d, 1H, CH-Ar), 6.98–6.91 (d, 2H, 2 × C*H*-Ar), 6.84–6.83 (d, 2H, 2 × C*H*-Ar), 6.72–6.67 (dd, 2H, 2 × C*H*-Ar), 4.41 (s, 2H, C*H*_2_–NH–C=S), 4.31–4.29 (d, 2H, CH_2_–C*H*_2_–O–C=O), 3.74–3.71 (t, 2H, C*H*_2_–CH_2_–O–C=O), 3.66–3.51 (m, 10H, 5 × C*H*_2_–O), 3.35–3.30 (m, C*H*_2_–N_3_ and MeOD solvent residual peak). HRMS (ESI/QTOF) *m*/*z*: [M + H]^+^ calcd for C_31_H_32_N_5_O_10_S^+^ 666.1864; found 666.1870.

#### Analytical data for alkyne-cleavable-FITC

8.27 (m, 1H, C*H*-Ar), 7.90–7.88 (d, 1H, C*H*-Ar), 7.24–7.22 (d, 1H, C*H*-Ar), 6.89–6.87 (d, 2H, 2 × C*H*-Ar), 6.83–6.82 (d, 2H, 2 × C*H*-Ar), 6.70–6.68 (dd, 2H, 2 × C*H*-Ar), 4.43 (s, 2H, C*H*_2_–NH–C=S), 4.33–4.31 (t, 2H, CH_2_–C*H*_2_–O–C=O), 4.17–4.16 (d, 2H, CH_2_–C≡CH), 3.76–3.74 (m, 2H, C*H*_2_–CH_2_–O–C=O), 3.68–3.62 (m, 12H, 3 × C*H*_2_–CH_2_–O, 3 × CH_2_–C*H*_2_–O), 2.83–2.81 (t, 1H, C≡C–*H*). HRMS (ESI/QTOF) *m*/*z*: [M + H]^+^ calcd for C_34_H_35_N_2_O_11_S^+^ 679.1956; found 679.1967.

### Surface functionalization

#### Preparation of S-alkyne and S-azide slides

S-OH slides were exposed to oxygen plasma for 17 min. The substrates were transferred to a glass tube containing a 4.8 mM (1.5 mg mL^−1^) solution of APTES-alkyne (synthesis procedure described in ESI, Section 2.2†) in anhydrous toluene under nitrogen atmosphere, with or without addition of valeric acid (3.7 mM). The slides were incubated for 48 h at ambient temperature, washed with toluene and acetonitrile, and dried with a stream of nitrogen. Then, the slides were heated at 80 °C for 1.5 h and, if required, S-alkyne slides were stored under argon at −20 °C until further use. The preparation of S-azide slides was carried out according to the same protocol using a 12 mM (3 mg mL^−1^) solution of APTES-azide (synthesis procedure described in ESI, Section 2.2†).

#### Preparation of S-DIBO slides

S-OH slides were exposed to oxygen plasma for 17 min. The substrates were transferred to a glass tube containing a 1 mg mL^−1^ solution of APTES-DIBO in anhydrous toluene under nitrogen atmosphere, with the addition of valeric acid at a concentration of 3.7 mM. The slides were incubated for 48 h at ambient temperature, washed with toluene and acetonitrile, and dried with a stream of nitrogen. The slides were heated at 80 °C for 1.5 h and, if required, S-DIBO slides were stored under argon at −20 °C until further use.

#### Peptide conjugation *via* CuAAC (S-alkyne-P and S-azide-P)

The protocol refers to the conditions of Table 2, entries 9 and 13.

S-Alkyne (or S-azide) slides were immersed in a fresh solution of P-azide (or P-alkyne) in H_2_O/MeOH (1/1 v/v, 3.6 mM). CuSO_4_ (10 mM in H_2_O, 74 μL), THPTA (10 mM in H_2_O, 147 μL) and sodium ascorbate (14.6 mM in H_2_O, 250 μL) were sequentially added. The reaction mixture was degassed by bubbling argon for 7 min and shaken for 4 h at 25 °C. The resulting S-alkyne-P and S-azide-P slides were washed with Milli-Q water (3 times) and finally rinsed thoroughly with acetonitrile and dried with a stream of nitrogen.

#### Peptide conjugation *via* SPAAC

The protocol refers to the conditions of Table 3, entry 3. S-DIBO slides were immersed in a fresh solution of P-azide in MeOH (3.6 mM). The reaction mixture was degassed by bubbling argon for 7 min and shaken for 4 h at 25 °C. The resulting S-DIBO-P slides were washed with MeOH and MilliQ (3 times each) and used directly for the next step.

#### DNA immobilization

The protocol for functionalization of peptide-modified slides was adapted from our previously reported procedure.^28^ A peptide-functionalized slide (1 cm^2^) was immersed into a 2-(*N*-morpholino)ethanesulfonic acid (MES) solution (0.1 M, 2 mL) of EDC·HCl (50 mM) and HOBt (60 mM). Then, the NH_2_-DNA strand (5′-NH_2_-C6-AACAGCAAGAAGTGCAACGCCAAC) was added (1 nmol) and the reactor was shaken for 20 h at 25 °C, 750 rpm. The slide was rinsed with Milli-Q water and transferred in a new tube for washing with Tween 20 (0.1%, 10 mL), for 10 minutes. This operation was repeated twice to remove any residual unreacted DNA strand. The resulting slides were rinsed with MilliQ and stored in MilliQ at 4 °C until further use.

### Surface characterization

#### Quantification of alkyne, azide and DIBO moieties on silanized slides

The following method used to quantify the amount of alkyne, azide and DIBO at the surface of the silanized slides was adapted from Miyahara *et al.*,^31^ (Fig. S24†). A S-alkyne (or S-azide) slide was immersed in 2 mL of MeOH/ddH_2_O containing CuSO_4_ (0.015 μmol), sodium ascorbate (0.03 μmol) and 0.1 mg of N_3_-cleavable-FITC (or alkyne-cleavable-FITC (0.15 μmol)). The vessel was shaken under argon at 25 °C for 4 h. The slide was sequentially washed with Milli-Q water (3 times) and MeOH (3 times), under dark conditions. It was then immersed in 2 mL of 0.1 M NaOH aqueous solution, for 1 h, to cleave the fluorophore. The concentration of fluorophores was determined by measuring the fluorescence intensity of the bulk solution (*λ*_exc_ = 475 nm, *λ*_em_ = 525 nm). Quantification of DIBO moieties on the surface was done according to the same protocols using MeOH as solvent and without CuSO_4_ and sodium ascorbate.

#### Quantification of hybridization density

Quantification of the hybridization density was performed following our previously reported procedure.^28^ A DNA-modified slide was immersed in 1.5 mL of SSC 4× buffer containing 100 μL of a 10 μM Cy3-complementary reverse probe (5′Cy3-GTTGGCGTTGCACTTCTTGCTGTT) for 1.5 h at 25 °C. After hybridization, the slide was rinsed with Milli-Q water (5 times) and transferred in a new glass tube. The slide was then washed with 10 mL of 0.1% Tween-20 (3 times) for 10 minutes, 25 °C, 750 rpm, in order to remove non-hybridized Cy3-complementary reverse probe from the surface and finally rinsed with Milli-Q water (3 times).

The hybridized slide was immersed in 2 mL PBS 0.1× and heated at 85 °C for 17 min. The solution was collected and the fluorescence measured (*λ*_exc_ = 532 nm, *λ*_em_ = 568 nm). Hybridization density was determined using a calibration curve made with known concentration of Cy3-complementary reverse probe (Fig. S30†). The slide modified only with peptide was used as negative control in all experiments.

## Conclusions

In summary, we demonstrated the feasibility of using branched spacers based on peptides as anchoring units to immobilize DNA probes on glass slides. The conjugation strategy, relying on sequential silanization and azide to alkyne [3 + 2] cycloaddition click reactions was probed by XPS measurements and fluorescence-based quantification assays of intermediate surface reactive functionalities and final DNA hybridization density. Both copper-catalyzed and copper-free click reactions for peptide conjugation on cross-reactive silanized slides resulted in a high DNA hybridization density, above 2 pmol cm^−2^.

Furthermore, variation on the microstructure of the peptide units would be an interesting aspect of using of peptide-derived anchors,^37,38^*e.g.* for tuning the space between side chain functional groups in peptide sequence or modifying the folding behaviour of peptide architectures by introducing triazole moieties.^39^ Such parameters could have an effect on steric hindrance and orientation of immobilized DNA probes and consequently modulate the hybridization efficiency of target DNA. Further perspectives of peptide-based biosensors include the modulation of surface properties such as the introduction of antifouling capacities or charged layers by proper selection of specific amino acids.

## Author contributions

Alireza Kavand: conceptualization, investigation, methodology, validation, data curation, writing – original draft, writing – review & editing. Perrine Robin: conceptualization, investigation, methodology, validation, data curation, writing – original draft, writing – review & editing. Lucas Mayoraz: investigation, methodology. Mounir Mensi: methodology. Sandrine Gerber-Lemaire: funding acquisition, supervision, project administration, writing – original draft, writing – review & editing.

## Conflicts of interest

The authors declare no competing financial interest.

## Supplementary Material

RA-013-D3RA04928K-s001
