# Supplementary material for: Achieving high hybridization density at DNA biosensor surfaces using branched spacer and click chemistry
Source: RSC Adv. 2023 Nov 21;13(48):34003–11. doi: 10.1039/d3ra04928k (PMC10660212; doi:10.1039/d3ra04928k)
Supplement: RA-013-D3RA04928K-s001 [file RA-013-D3RA04928K-s001.pdf]

Electronic Supplementary Material (ESI) for RSC Advances.
This journal is © The Royal Society of Chemistry 2023

Supporting Information

Achieving high hybridization density at DNA biosensor surfaces using branched spacer and click chemistry.

Alireza Kavand[a], Perrine Robin[a], Lucas Mayoraz[a], Mounir Mensi[b], Sandrine Gerber-Lemaire[a,*]

[a] Group for Functionalized Biomaterials, Institute of Chemical Sciences and Engineering, Ecole Polytechnique Fédérale de Lausanne, CH-1015 Lausanne, Switzerland
[b] ISIC-XRDSAP, EPFL Valais-Wallis, Rue de l'Industrie 17, CH-1951, Sion, Switzerland
* Corresponding author.
E-mail address: sandrine.gerber@epfl.ch (S. Gerber-Lemaire)

Table of content

1. General 2
1.1. Materials 2
1.2. Characterizations 3
2. Synthesis 4
2.1 Peptide synthesis 4
2.2 Synthesis of silanization reagents 13
2.2.1. Synthesis of 3-azidopropyltriethoxysilane (APTES-N_3) 13
2.2.2. Synthesis of N-(3-(triethoxysilyl)propyl)hex-5-ynamide (APTES-alkyne) 13
2.2.3. Synthesis of APTES-DIBO 14
2.3. Synthesis of fluorescent labelling reagents 22
2.3.1. Synthesis of N_3-cleaveable-FITC 22
2.3.2. Synthesis of alkyne-cleavable-FITC 26
3. Surface characterization 30
3.1. Quantification of surface reactive groups after silanization 30
3.2. XPS analysis of functionalized slides 31
3.2.1. Characterization of silanized surfaces 31
3.2.2. Characterization of peptide conjugated surfaces 31
3.2.3. Characterization of DNA functionalized surfaces 33
3.3. Stability of DNA functionalized slides upon storage at 4°C 35

1. General

1.1. Materials

Chlorotrityl chloride resin (1-1.6 mmol/g, 100-200 mesh) was purchased from abcr GmbH.
1-Hydroxybenzotriazole hydrate (HOBt, ≥97%), anhydrous N,N-dimethylformamide (anhydrous DMF, 99.8%), anhydrous dichloromethane (anhydrous DCM, ≥99.8%), N,N-dimethylformamide (DMF, >99%), trifluoroacetic acid (TFA, 99%), anhydrous N-Ethyldiisopropylamine (DIPEA, 99%), N-(3-Dimethylaminopropyl)-N′-ethylcarbodiimide–hydrochloride (EDC.HCl, 98%), Tris(3-hydroxypropyltriazolylmethyl)amine (THPTA, 95%), 5-hexynoic acid (97%), piperidine (99%), MES hydrate (≥99.5%), fluorescein isothiocyanate isomer I (FITC, >90%), copper(II) sulfate anhydrous ($CuSO_4$, ≥99.99% trace metals basis), tetraethylene glycol, 4-(dimethylamino)pyridine (DMAP, ≥99%), N,N'-dicyclohexylcarbodiimide (DCC, 99%), propargyl bromide (80% in toluene) and diethyl ether (DEE, 99%) were purchased from Sigma-Aldrich. Sodium ascorbate (NaAsc, ≥98%) was purchased from Carl Roth GmbH. N,N,N′,N′-tetramethyl-O-(1H-benzotriazol-1-yl)uronium hexafluorophosphate (HBTU, 99%), 2,2,2-trifluoroethanol (TFE, 99%), tetrabutylammonium bromide (TBAB, 99%) and saline-sodium citrate buffer (SSC 20X, pH 7.0) were purchased from Alfa Aesar. Tosyl chloride (99%) and N-(tert-butoxycarbonyl)glycine (>98.0%) were purchased from TCI. Fmoc-Glu(OtBu)-OH (99%) and NaH (60% dispersion in mineral oil) were purchased from Fluorochem. Triphenylphosphine (99%), sodium azide (NaN_3, 99%), sodium thiosulfate ($Na_2S_2O_3$), bromine (>99.5%, ACS reagent), anhydrous dioxane (molecular sieves, 99.5%, stabilized), and 4-nitrophenyl chloroformate (97%) were purchased from Acros Organics. Methanol (MeOH, 99.8%), acetonitrile (ACN, >99.9%), ethanol (absolute, 99.9%), acetone (99.8%), phenylacetaldehyde (98%, stabilized), trimethylsilyliodide (97%, stab. with copper), n-butyllithium (n-BuLi, 1.6 M in hexane), diisopropylamide (LDA, redistilled, 99.5%), and 3-aminopropyltriethoxysilane (APTES, 99%) were purchased from Thermo Fisher Scientific. Phosphate-Buffered Saline (PBS 1X, pH 7.4) was purchased from Gibco. Single-stranded oligonucleotides (ssDNA) were purchased from GenScript with HPLC purification and have the following sequences: 5'AmMC6-AACAGCAAGAAGTGCAACGCCAAC and 5'Cy3-GTTGGCGTTGCACTTCTTGCTGTT as probe and Cy3 tagged complementary strand, respectively. Magnesium sulfate ($MgSO_4$) was purchased from Reactolab. Borosilicate glass slides (10 mm × 10 mm × 0.5 mm, ISO class 5 clean room production) were purchased from SCHOTT AG.

6-Azidohexanoic acid was synthesized as reported in literature (D. Chan-Seng and J. F. Lutz, *ACS Macro Letters*, 2014, **3**, 291–294). The Kaiser test was carried out as described in the literature (E. Kaiser, R. L. Colescott, C. D. Bossinger and P. I. Cook, Analytical Biochemistry, 1970, 34, 595–598). Solid phase extraction (SPE) tubes (50 and 20 ml polypropylene SPE tubes with polyethylene frits, 20 µm porosity purchased from SUPELCO®) were used for peptide preparation on solid support. All coating procedures were conducted with a MSC-100 Cooling Thermoshaker Incubator from Labgene Scientific, Switzerland. The glass tubes (reactors) for coating procedures were cleaned before use by immersion in a solution of 1 M of NaOH for overnight, rinsed with distilled water and acetone, and dried in an oven. The slide surfaces were cleaned and activated with a Femto O_2 Plasma system (200 W, Diener Electronic). All aqueous solutions were prepared with Milli-Q water and all chemicals were used as received, except if noted otherwise.

1.2. Characterizations

X-ray Photoelectron Spectroscopy (XPS) measurements were carried out on an Axis Supra (Kratos Analytical) using the monochromated Ka X-ray line of an Aluminium anode at the X-Ray Diffraction and Surface Analytics Platform (EPFL–ISIC–XRDSAP, Sion, Switzerland). The pass energy was set to 40 eV with a step size of 0.15eV. Charge neutralization was done using a low energy electron gun and the spectra were referenced at 284.8 eV using the aliphatic component of the C 1s orbital. The XPS spectra processing (e.g. Gaussian peak fitting and elements present on the surface) was carried out by a commercial CasaXPS software package using a Shirley and linear background (Casa Software Ltd., U.K.).
Fluorescence measurements were carried out with a Synergy H1 microplate reader from BioTek.
^{1}H and ^{13}C NMR spectra were recorded in $CDCl_3$, MeOD, or D_2O on Bruker Avance III-400, Bruker Avance-400 or Bruker DRX-400 spectrometers (Bruker, Billerica, MA, USA).
The qualitative accurate masses were measured by ESI-TOF using the Xevo G2-S QTOF (Waters) and nanoESI-FT-MS using the Elite™ Hybrid Ion Trap-Orbitrap (ThermoFisher) Mass Spectrometer.

2. Synthesis

2.1 Peptide synthesis

[Glu)]$_3$-azide, [Glu)]$_3$-alkyne, Met.[Glu)]$_2$-azide and Met.[Glu)]$_2$-alkyne were synthetized via solid-phase peptide synthesis, following the steps described below.

Preparation of CTC-[Glu(O^{t}Bu)]$_3$

Loading of the first amino acid on the resin. 0.5 g of chlorotrityl chloride resin (≈ 0.8 mmol, 1 equiv) was swollen in 5 ml of anhydrous DCM and washed 3 times with 3 mL of anhydrous DCM in a 50 ml SPE tube under argon. Fmoc-Glu(O^{t}Bu)-OH (2 equiv, 0.681 g, 1.6 mmol) was added and the tube was then sealed by rubber sealing stopper (for loading of Fmoc-Met-OH, 0.594 g was added). The vessel was degassed by performing 3 vacuum purging/argon cycles. Anhydrous DCM (4 mL) was added and the tube was agitated on a shaker for 10 min in order to complete dissolving of amino acid and swelling of resin under argon. DIPEA (4 equiv, 0.6 mL, 3.2 mmol) was added and the mixture was agitated for 1.5 h under argon at r.t. The solution was filtered and the resin was washed 6 times with DMF and DCM. In order to cap unreacted groups on the resin, a mixture of DCM, methanol, and DIPEA (0.8, 1.5, 0.5 mL, respectively) was added and the tube was agitated for 15 min (twice). The solution was filtered, and the resin was washed 6 times with DMF and DCM, respectively. The resin was dried under vacuum at room temperature for 48 h. The yield of amino acid grafted on the resin was measured by gravimetric analysis, and found to be 1.12 mmol/g (70 %).

Successive couplings of Glu (O^{t}Bu)-OH. The Fmoc-protecting groups were removed by agitation of the resin for 5 min with 10 mL of 25% (vol/vol) piperidine solution in DMF. After filtering and rinsing the resin with DMF, the deprotection procedure was carried out again for a duration of 30 minutes. Subsequently, the resin was washed with DMF and DCM. Fmoc-Glu(OtBu)-OH (0.70 g, 1.65 mmol, 3 equiv), HBTU (3 equiv, 0.63 g, 1.65 mmol) and HOBt (3 equiv, 0.22 g, 1.65 mmol) were dissolved in 5 ml anhydrous DMF and added to the tube. DIPEA (0.6 mL, 3.3 mmol, 6 equiv) was added to the solution, which was transferred to the SPE tube. The mixture was agitated for 1.5 h at r.t. After filtration and washing of the resin, completion of the coupling was monitored using the Kaiser test. The procedure was repeated for coupling another Glu(O^{t}Bu)-OH.

Preparation of CTC-[Glu(O^{t}Bu)]$_3$-azide

6-Azidohexanoic acid (3 equiv, 0.280 g, 1.65 mmol), HBTU (3 equiv, 0.63 g, 1.65 mmol) and HOBt (3 equiv, 0.22 g, 1.65 mmol) were dissolved in 5 mL of anhydrous DMF. DIPEA (6 equiv, 0.6 mL, 3.3 mmol) was added to the solution, which was transferred to the SPE tube. The mixture was agitated for 1.5 h at r.t. After filtration and washing of the resin, completion of the coupling was monitored using the Kaiser test.

Preparation of CTC-[Glu(O^{t}Bu)]$_3$-alkyne

5-Hexynoic acid (3 equiv, 0.176 g, 1.57 mmol), HBTU (3 equiv, 0.59 g, 1.57 mmol) and HOBt (3 equiv, 0.21 g, 1.57 mmol) were dissolved in 5 mL of anhydrous DMF. DIPEA (6 equiv, 0.4 mL, 3.15 mmol) was added to the solution, which was transferred to the SPE tube. The mixture was agitated for 1.5 h at r.t. After filtration and washing of the resin, completion of the coupling was monitored using the Kaiser test.

Cleavage from the resin

The resin was washed with methanol (3 times) and DCM (6 times), and transferred to a glass vessel. A solution of DCM/TFE (80:20) was added and the mixture was agitated for 45 min at r.t. The solution was collected by filtration. Treatment of the resin with a solution of DCM/TFE (80:20) and filtration was repeated twice. The collected solutions were combined and concentrated under vacuum. The resulting peptide was obtained by precipitation in DEE/hexane (1:1) and dried under vacuum at r.t. for 48 h.

For the preparation of **Met.[Glu(O*t*Bu)]$_2$-azide** and **Met.[Glu(O*t*Bu)]$_2$-alkyne**, the above procedure is applied with the loading of Fmoc-Met-OH to the CTC resin as first amino acid residue.

[Glu(O*t*Bu)]$_3$-azide: ^{1}H NMR (400 MHz, MeOD) δ 4.39 – 4.20 (m, 3H), 3.19 (d, J = 6.8 Hz, 3H), 2.32 – 2.13 (m, 8H), 2.13 – 1.90 (m, 3H), 1.90 – 1.68 (m, 3H), 1.65 – 1.45 (m, 4H), 1.35 (d, J = 1.5 Hz, 29H). ^{13}C NMR (101 MHz, MeOD) δ 174.74, 173.14, 172.63, 172.52, 172.36, 172.30, 172.08, 80.44, 80.38, 80.35, 52.56, 52.38, 51.50, 50.90, 35.16, 31.27, 31.24, 31.04, 28.23, 27.05, 26.98, 26.82, 26.49, 25.97, 24.95. HRMS (ESI/QTOF) m/z: [M + Na]$^+$ Calcd for $C_{33}H_{56}N_6NaO_{11}^+$ 735.390478; Found 735.38993. ^{1}H NMR and ESI-MS spectra are given in Figure S1.

[Glu(O*t*Bu)]$_3$-alkyne: ^{1}H NMR (400 MHz, MeOD) δ 4.38 – 4.19 (m, 3H), 2.33 – 2.19 (m, 8H), 2.17 – 1.92 (m, 6H), 1.88 – 1.65 (m, 5H), 1.43 – 1.26 (m, 26H). ^{13}C NMR (101 MHz, MeOD) δ 174.16, 173.13, 172.65, 172.54, 172.38, 172.31, 172.11, 82.79, 80.45, 80.39, 80.37, 68.90, 52.60, 52.38, 51.48, 34.14, 31.24, 31.04, 27.00, 26.78, 26.49, 24.37, 17.28. HRMS (ESI/QTOF) m/z: [M + Na]$^+$ Calcd for $C_{33}H_{53}N_3NaO_{11}^+$ 690.3572; Found 690.3582. ^{1}H NMR and ESI-MS spectra are given in Figure S2.

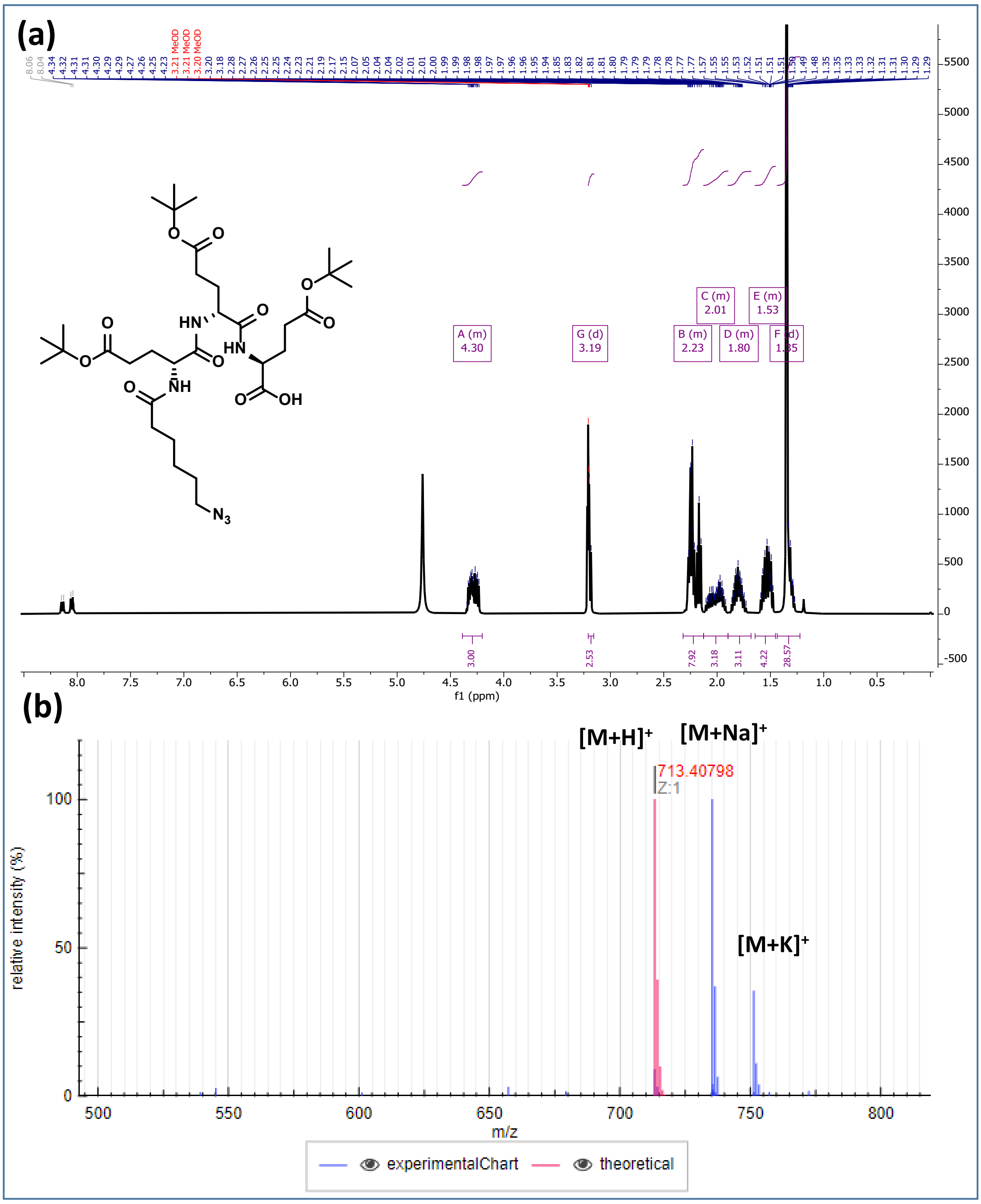

Figure S1. *^{1}H NMR spectrum of [Glu(O^{t}Bu)]$_3$-azide (a), ESI-MS spectrum of [Glu(O^{t}Bu)]$_3$-azide (b).*

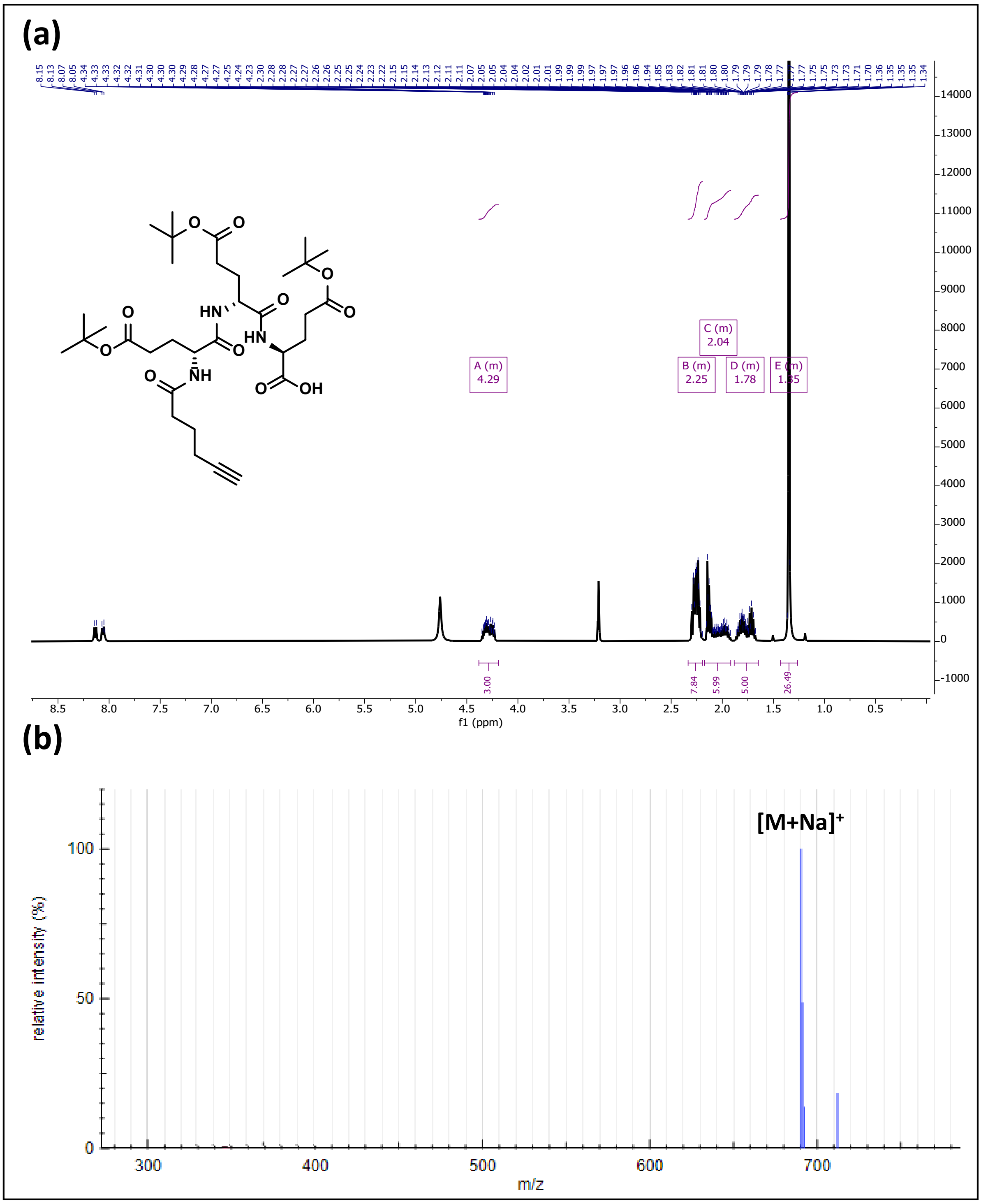

Figure S2. *^{1}H NMR spectrum of [Glu(O^{t}Bu)]$_3$-alkyne (a), ESI-MS spectrum of [Glu(O^{t}Bu)]$_3$-alkyne (b).*

Met.[Glu(O^{t}Bu)]$_2$-azide. ^{1}H NMR (400 MHz, CDCl$_3$) δ 4.59 – 4.29 (m, 3H), 3.21 (t, *J* = 6.9 Hz, 2H), 2.59 – 2.42 (m, 2H), 2.39 – 2.10 (m, 7H), 2.09 – 1.80 (m, 8H), 1.57 (dp, *J* = 21.8, 7.3 Hz, 4H), 1.38 (d, *J* = 2.2 Hz, 19H). ^{13}C NMR (101 MHz, CDCl$_3$) δ 173.91, 173.61, 173.43, 173.36, 171.81, 171.57, 81.33, 81.29, 77.24, 53.57, 53.00, 52.12, 51.21, 36.15, 31.76, 31.69, 30.65, 30.10, 28.57, 28.08, 28.06, 27.41, 27.17, 26.34, 24.89, 15.29. HRMS (ESI/QTOF) m/z: [M + Na]$^+$ Calcd for $C_{29}H_{50}N_6NaO_9S^+$ 681.3252; Found 681.3267.

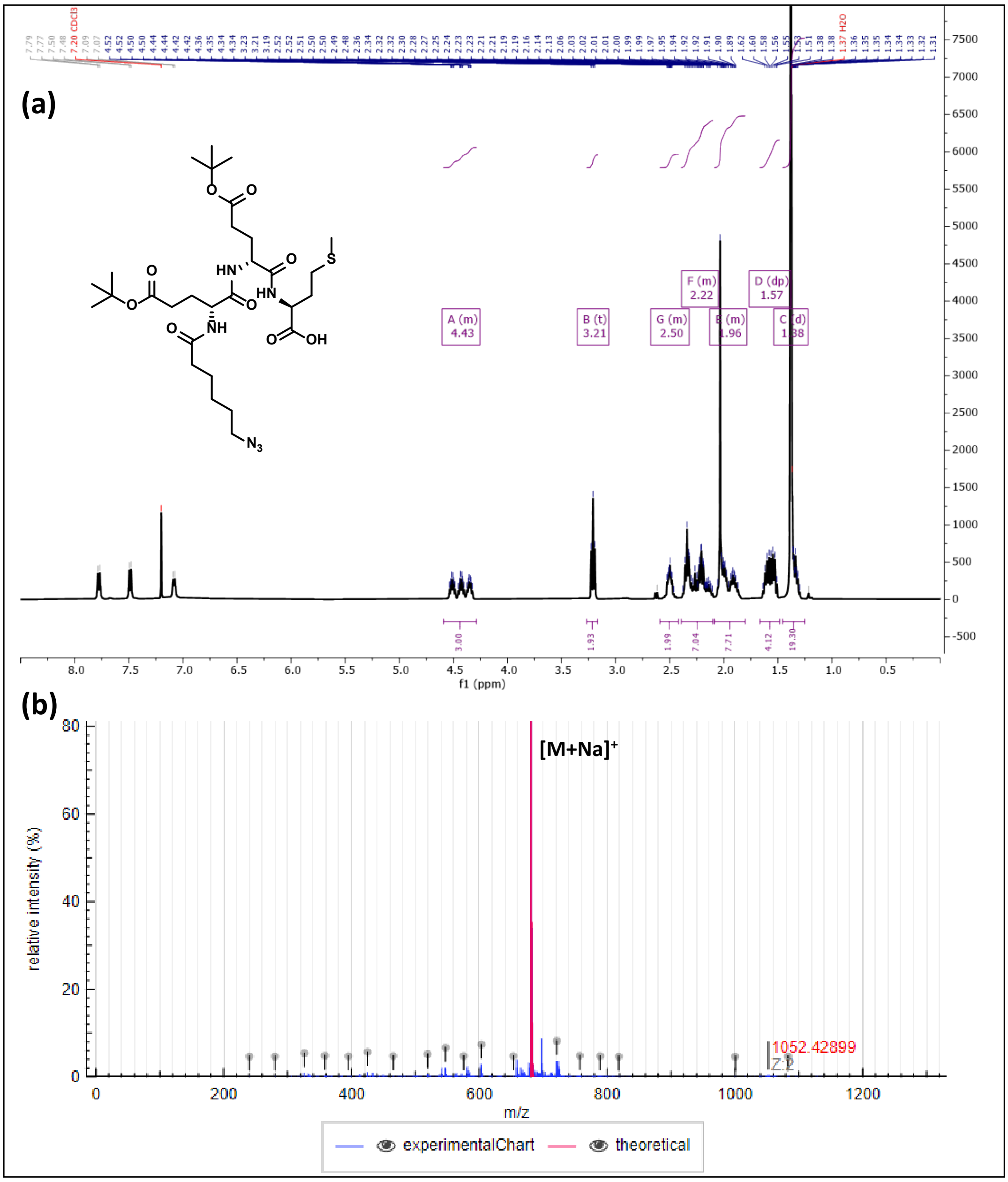

Figure S3. *^{1}H NMR spectrum of Met.[Glu(O^{t}Bu)]$_2$-azide (a), ESI-MS spectrum of Met.[Glu(O^{t}Bu)]$_2$-azide (b).*

Met.[Glu(O^{t}Bu)]$_2$-alkyne.^{1}H NMR (400 MHz, $CDCl_3$) δ 4.58 – 4.31 (m, 3H), 2.50 (td, *J* = 7.9, 5.1 Hz, 2H), 2.41 – 2.22 (m, 6H), 2.22 – 2.08 (m, 3H), 2.07 – 1.84 (m, 9H), 1.84 – 1.72 (m, 2H), 1.38 (d, *J* = 2.4 Hz, 17H). ^{13}C NMR (101 MHz, $CDCl_3$) δ 173.63, 173.45, 173.38, 173.31, 171.77, 171.62, 83.32, 81.37, 81.30, 77.23, 69.37, 53.59, 53.01, 52.14, 34.88, 31.73, 30.59, 30.10, 28.09, 28.07, 27.42, 27.16, 23.93, 17.90, 15.31. HRMS (ESI/QTOF) m/z: [M + Na]$^+$ Calcd for $C_{29}H_{47}N_3NaO_9S^+$ 636.2925; Found 636.2925.

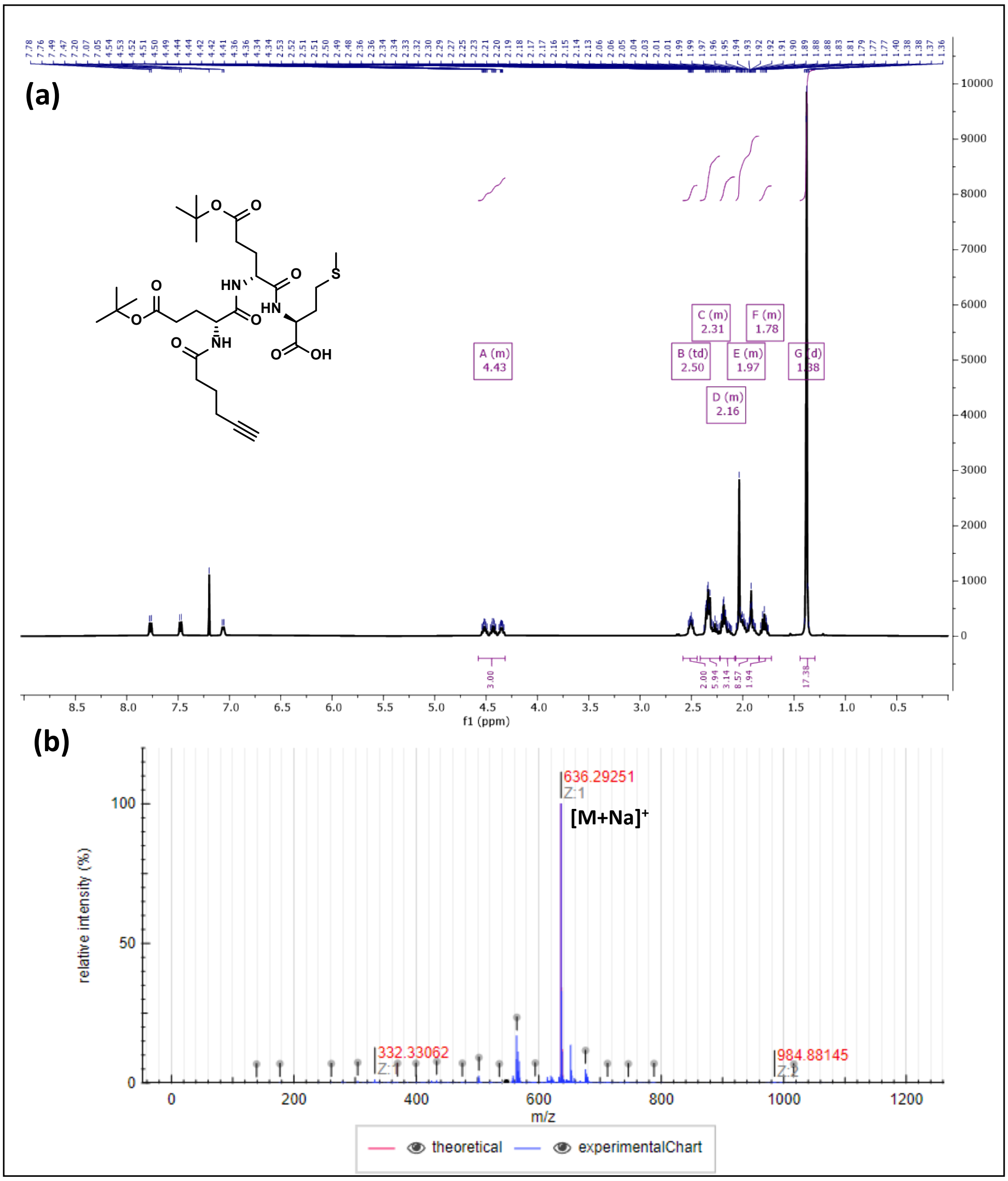

Figure S4. *^{1}H NMR spectrum of Met.[Glu(O^{t}Bu)]$_2$-alkyne (a), ESI-MS spectrum of Met.[Glu(O^{t}Bu)]$_2$-alkyne (b).*

Deprotection [Glu(O^{t}Bu)]$_3$-azide and [Glu(O^{t}Bu)]$_3$-alkyne (P-azide and P-alkyne)

The peptide was dissolved in a mixture of TFE (5 mL), DCM (5 mL) and anisole (2 mL) and the solution was stirred for 6 h at r.t. The solvents were evaporated and the product was precipitated in cold DEE/hexane (1:1) and the suspension was centrifuged. The supernatant was discarded and the resulting solid was dried under vacuum overnight. The solid product was dissolved in 10 mL of distilled water and freeze-dried to afford **P-azide** (86% yield) or **P-alkyne** (81% yield) as white solids.

P-azide. ^{1}H NMR (400 MHz, MeOD): δ 4.38 – 4.21 (m, 3H), 3.20 – 3.17 (m, 2H), 2.43 – 2.26 (m, 6H), 2.25 – 1.96 (m, 5H), 1.84 (dddd, J = 14.1, 8.9, 7.0, 4.9 Hz, 3H), 1.77 – 1.44 (m, 4H), 1.41–1.24 (m, 2H). HRMS (ESI/QTOF) m/z: [M + H$_{-1}$]$^-$ Calcd for $C_{21}H_{31}N_6O_{11}^-$ 543.2056; Found 543.2045.

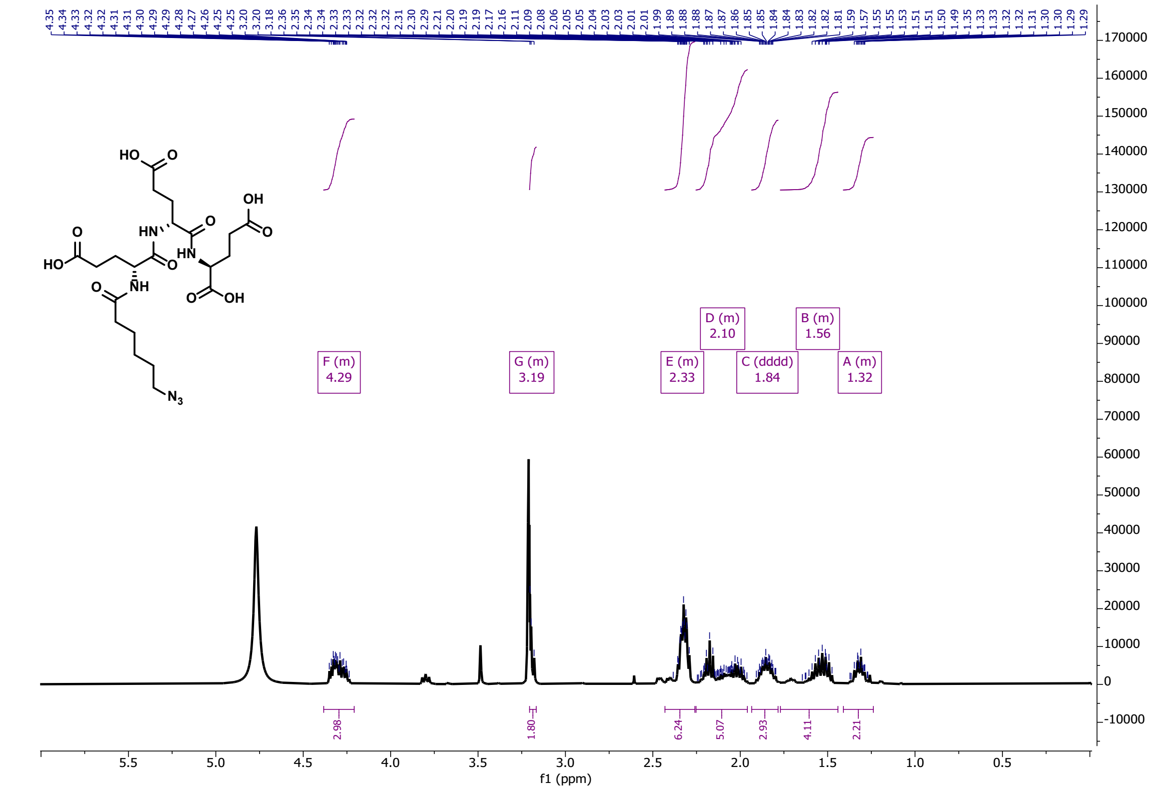

Figure S5. *^{1}H NMR spectrum of* ***P-azide***

P-alkyne. ^{1}H NMR (400 MHz, MeOD): δ 4.49 – 4.33 (m, 3H), 2.51 – 2.34 (m, 8H), 2.28 – 2.05 (m, 6H), 2.04 – 1.88 (m, 3H), 1.87 – 1.75 (m, 2H). HRMS (ESI/QTOF) m/z: $[M + H_{-1}]^-$ Calcd for $C_{21}H_{28}N_3O_{11}^-$ 498.1729; Found 498.1742.

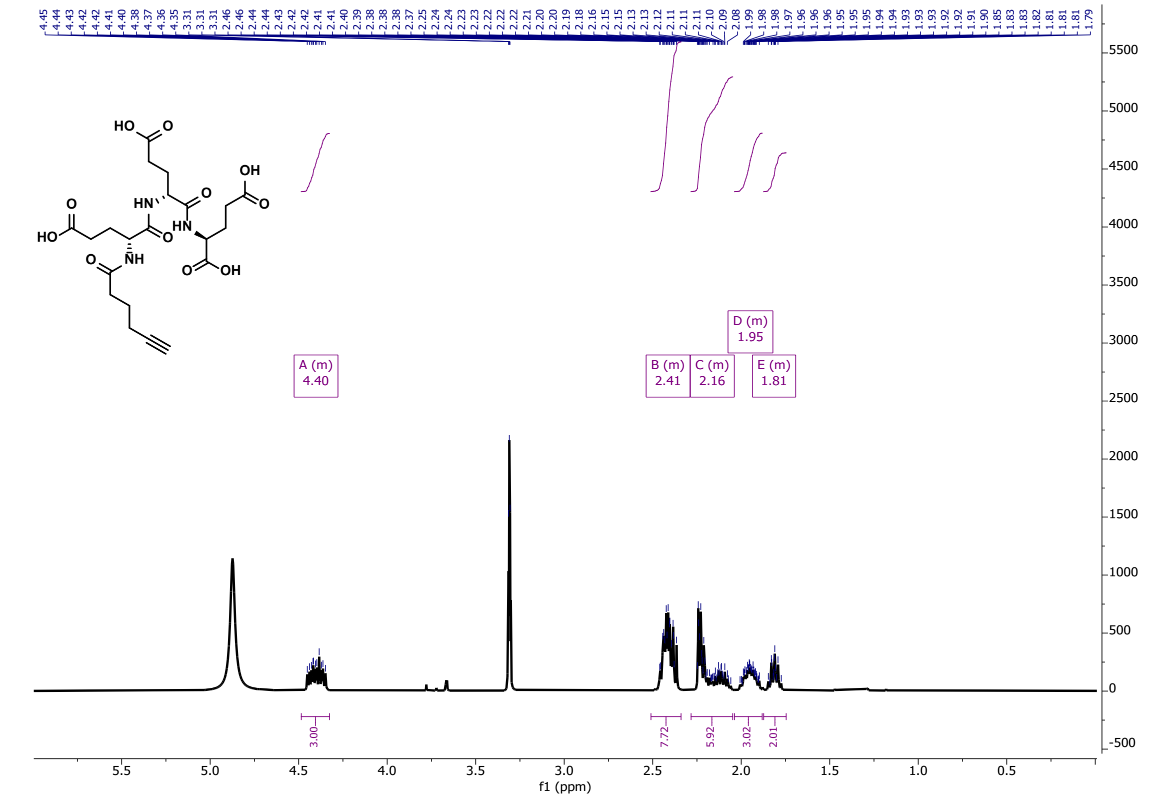

Figure S6. *^{1}H NMR spectrum of* ***P-alkyne***

The deprotection of **Met.[Glu(O^{t}Bu)]$_2$-azide** and **Met.[Glu(O^{t}Bu)]$_2$-alkyne** is performed with the same procedure to afford **Ps-azide** and **Ps-alkyne**, respectively.

Ps-azide. ^{1}H NMR (400 MHz, MeOD) δ 4.49 – 4.14 (m, 3H), 3.21 – 3.16 (m, 2H), 2.57 – 2.41 (m, 2H), 2.40 – 2.26 (m, 4H), 2.24 – 2.13 (m, 2H), 2.13 – 1.96 (m, 5H), 1.95 – 1.76 (m, 3H), 1.67 – 1.44 (m, 5H), 1.40 – 1.25 (m, 2H). HRMS (ESI/QTOF) m/z: $[M + H_{-1}]^-$ Calcd for $C_{21}H_{33}N_6O_9S^-$ 545.2035; Found 545.2035.

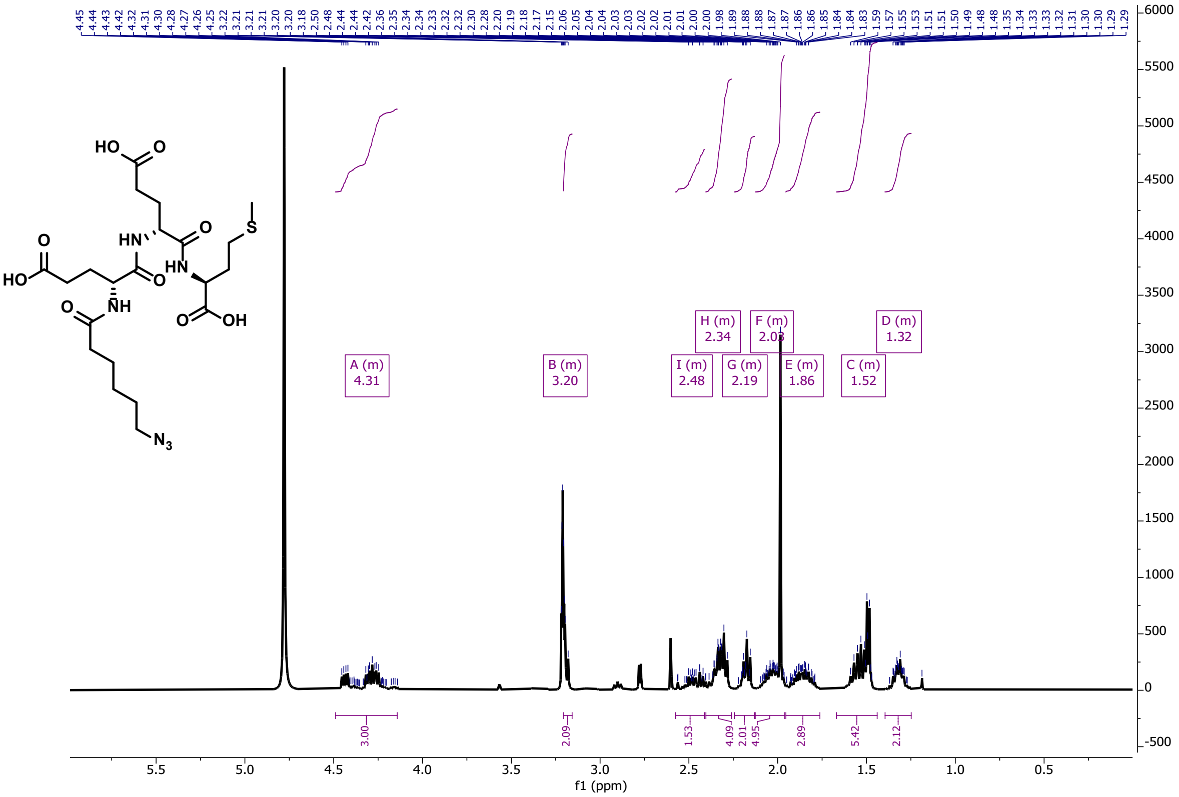

Figure S7. *^{1}H NMR spectrum of Ps-azide*

Ps-alkyne. ^{1}H NMR (400 MHz, MeOD) δ 4.49 – 4.11 (m, 3H), 2.58 – 2.24 (m, 7H), 2.18 – 2.10 (m, 3H), 2.09 – 1.95 (m, 5H), 1.94 – 1.78 (m, 3H), 1.78 – 1.64 (m, 2H). HRMS (ESI/QTOF) m/z: $[M + H_{-1}]^-$ Calcd for $C_{21}H_{30}N_3O_9S^-$ 500.1708; Found 500.1714.

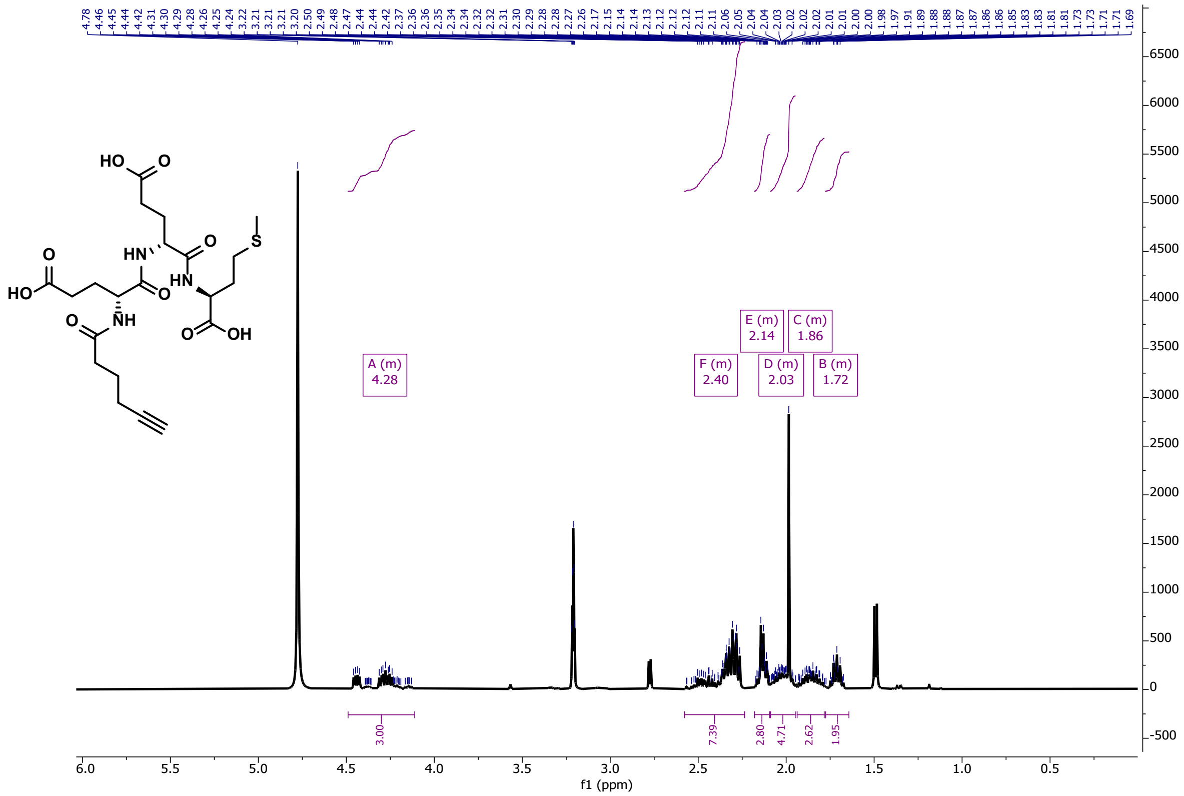

Figure S8. *^{1}H NMR spectrum of* ***Ps-alkyne***

2.2 Synthesis of silanization reagents

2.2.1. Synthesis of 3-azidopropyltriethoxysilane (APTES-N_3)

The preparation of **APTES-N_3** was adapted from a procedure reported in Kantheti, S.; Narayan, R.; Raju, K. VSN. Pyrene-anchored ZnO nanoparticles through click reaction for the development of antimicrobial and fluorescent polyurethane nanocomposite. *Polym. Int.* **2015**, *64*, 267-274.

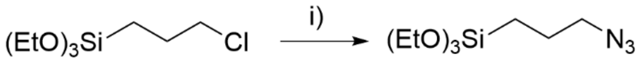

Scheme S1. *Synthesis of* ***APTES-N_3***. Conditions: NaN_3, Bu_4NBr cat., dry ACN, reflux, 24h.

NaN_3 (2.0 equiv, 1.08 g, 16.6 mmol) and tetrabutylammonium bromide (0.24 equiv , 0.64 g, 2 mmol) was dissolved in dry ACN (50 mL) under argon atmosphere. 3-Chloropropyltriethoxysilane (1.0 equiv, 2 g, 8.3 mmol) was added and the mixture was stirred under reflux for 24 h. The volatiles were evaporated. The crude product was diluted in DEE (50 mL) and the suspension was filtered and washed with DEE. The combined solutions were evaporated to afford **APTES-N_3** as a colorless liquid (1.85 g, 90 %). The analytical data were in accordance with reported characterizations.

^1H NMR (400 MHz, $CDCl_3$) δ 4.14 (q, J = 7.0 Hz, 6H), 3.58 (t, J = 7.0 Hz, 2H), 2.12 – 1.95 (m, 2H), 1.55 (t, J = 7.0 Hz, 9H), 1.08 – 0.93 (m, 2H).

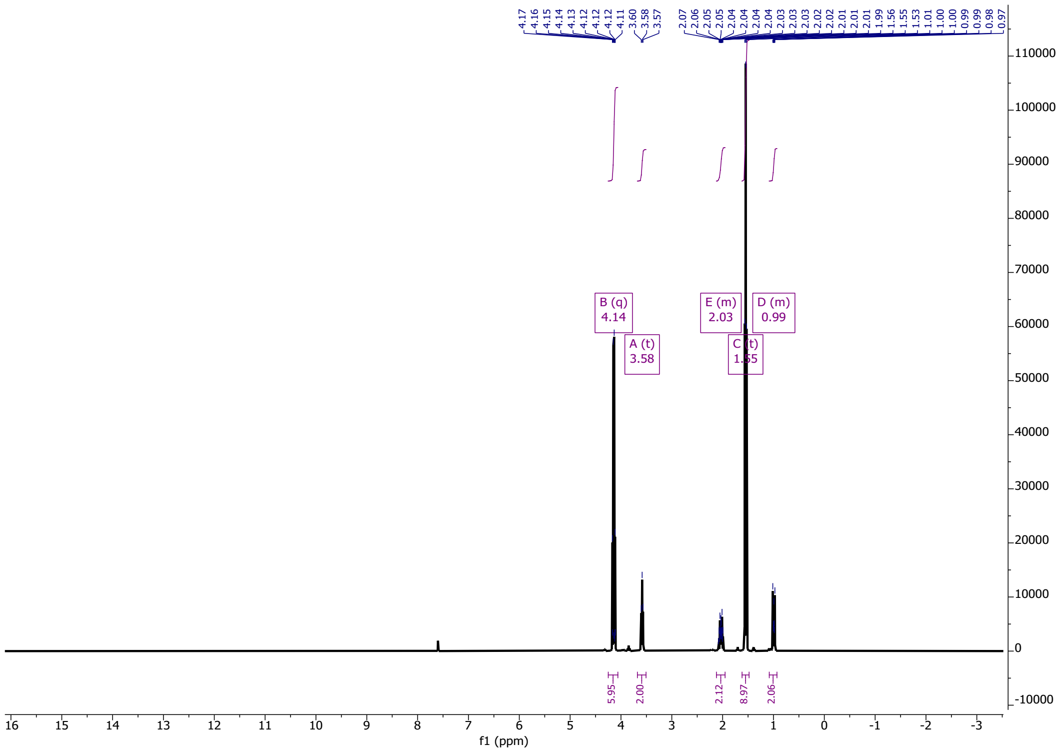

Figure S9. *^1H NMR spectrum of* ***APTES-N_3***

2.2.2. Synthesis of N-(3-(triethoxysilyl)propyl)hex-5-ynamide (APTES-alkyne)

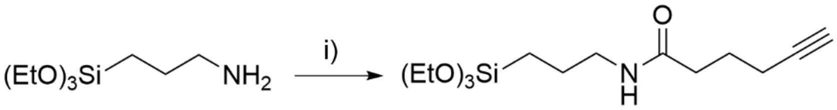

Scheme S2. *Synthesis of* ***APTES-alkyne***. Conditions: 5-hexynoic acid, EDC.HCl, DCM, 0°C to r.t, 4h.

5-Hexynoic acid, (1.0 equiv, 253 mg, 2.26 mmol) was dissolved in DCM (50 mL). EDC.HCl (1.0 equiv, 433 mg, 2.26 mmol) dissolved in DCM (10 mL) was then added dropwise. The solution was cooled to 0°C and stirred for 10 min. (3-Aminopropyl)triethoxysilane (1.0 equiv, 0.5 g, 2.26 mmol) dissolved in DCM (5 mL) was added dropwise and the reaction mixture was stirred at 0 °C for 2 hours, then at r.t. for 2 hours. The volatiles were evaporated under reduced pressure. The crude product was purified by flash column chromatography (silica gel, EtOAc/hexane (4:1)) to provide APTES-alkyne as a yellow oil (0.45 g, 63%).

^{1}H NMR (400 MHz, $CDCl_3$) δ 3.80 (q, J = 7.0 Hz, 6H), 3.23 (td, J = 6.9, 5.8 Hz, 2H), 2.32 – 2.19 (m, 4H), 1.94 (t, J = 2.6 Hz, 1H), 1.88 – 1.79 (m, 2H), 1.66 – 1.56 (m, 2H), 1.20 (t, J = 7.0 Hz, 9H), 0.66 – 0.57 (m, 2H). ^{13}C NMR (101 MHz, $CDCl_3$) δ 172.05, 83.60, 77.23, 69.08, 58.49, 58.45, 41.81, 35.18, 24.22, 22.90, 18.45, 18.31, 17.86, 7.77.

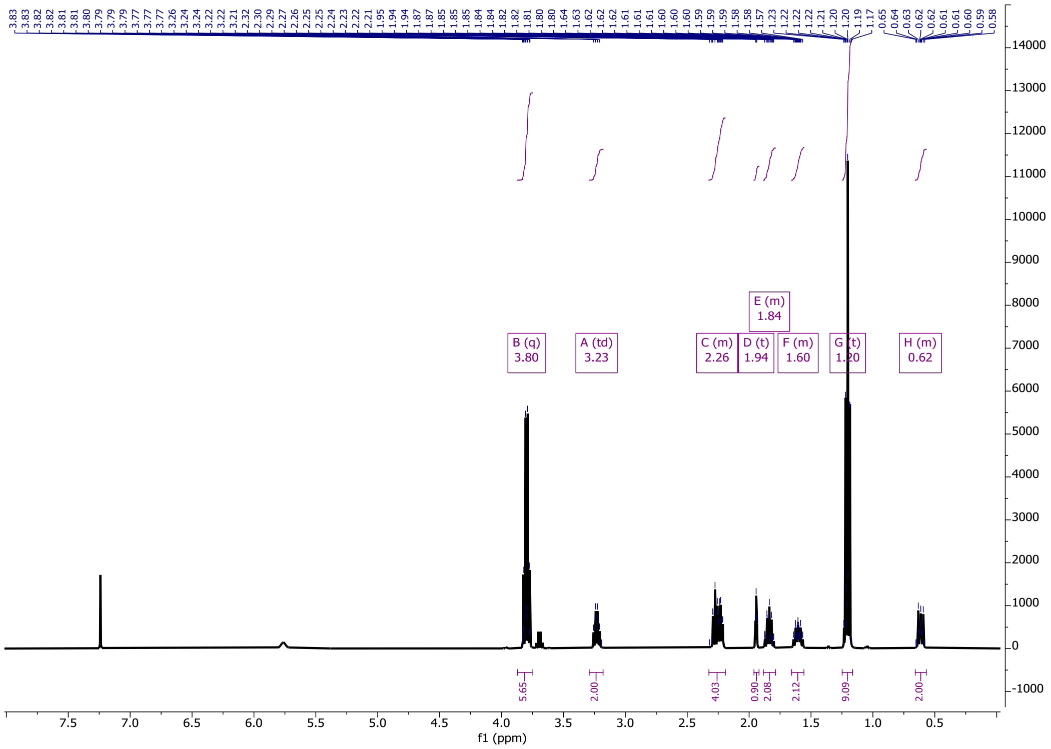

Figure S10. *^{1}H NMR spectrum of **APTES-alkyne***

2.2.3. Synthesis of APTES-DIBO

The synthesis of **APTES-DIBO** was performed following procedures adapted from: Mbua, N. E.; Guo, J.; Wolfert, M. A.; Steet, R.; Boons, G. J. Strain-promoted alkyne-azide cycloadditions (SPAAC) reveal new features of glycoconjugate biosynthesis. *ChemBioChem* **2011**, *12*, 1912–1921. Jung, M. E.; Miller, S. J. Total synthesis of isopavine and intermediates for the preparation of substituted amitriptyline analogs: facile routes to substituted dibenzocyclooctatrienes and dibenzocycloheptatrienes. *J. Am. Chem. Soc.* **1981**, *103*, 1984-1992. Jung, M. E.; Mossman, A. B.; Lyster, M. A. Direct synthesis of dibenzocyclooctadienes via double ortho Friedel-Crafts alkylation by the use of aldehyde-trimethylsilyl iodide adducts. *J. Org. Chem.* **1978**, *43*, 3698-3701.

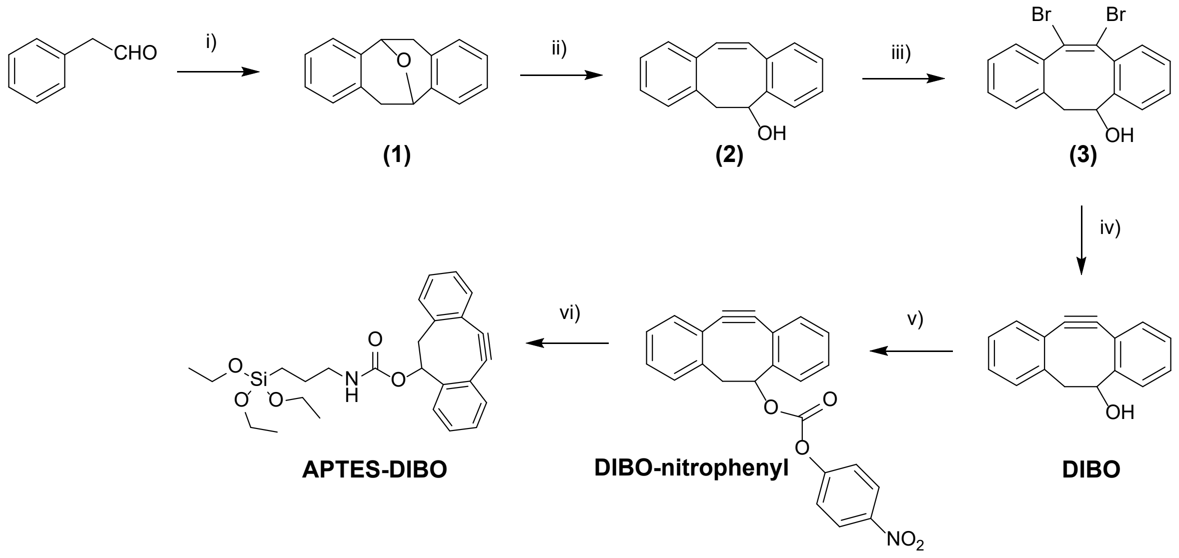

Scheme S3. *Synthesis of* ***APTES-DIBO***. Conditions: i) Phenylacetaldehyde, trimethylsilyl iodide, dry chloroform, 5°C, 7 days; ii) BuLi, dry THF, r.t, 4h; iii) Bromine, dry chloroform, r.t, 4h; iv) Lithium diisopropylamide, dry THF, r.t, 1h; v) 4-nitrophenylchloroformate, pyridine, dry DCM, r.t, 16h; vi) APTES, dry dioxane, r.t, overnight.

Synthesis of intermediate (1)

Phenyalcetaldehyde (1.0 equiv, 83.2 mmol, 9.7 mL) was dissolved in dry chloroform (40 mL) under inert conditions. The solution was cooled to 0°C. Trimethylsilyl iodide (1.02 equiv, 84.9 mmol, 12.1 mL) was added. The resulting mixture was stirred at 5°C for 7 days. The solution was quenched with saturated $Na_2S_2O_3$ (60 mL). The crude product was purified via column chromatography (silica gel, hexane:EtOAc 50:1 to 1:1) to afford **(1)** as a brown solid (25.2 mmol, 5.6 g, 86%). The analytical data were in accordance with previously reported data.

^{1}H NMR (400 MHz, $CDCl_3$) δ 7.15-7.07 (m, 6H, 6 × Ar-*H*), 6.99-6.97 (m, 2H, 2 × Ar-*H*), 5.31-5.29 (d, 2H, 2 × C*H*), 3.56 (dd, 2H, 2 × HC-*H*), 2.80 (d, 2H, 2 × HC-*H*).

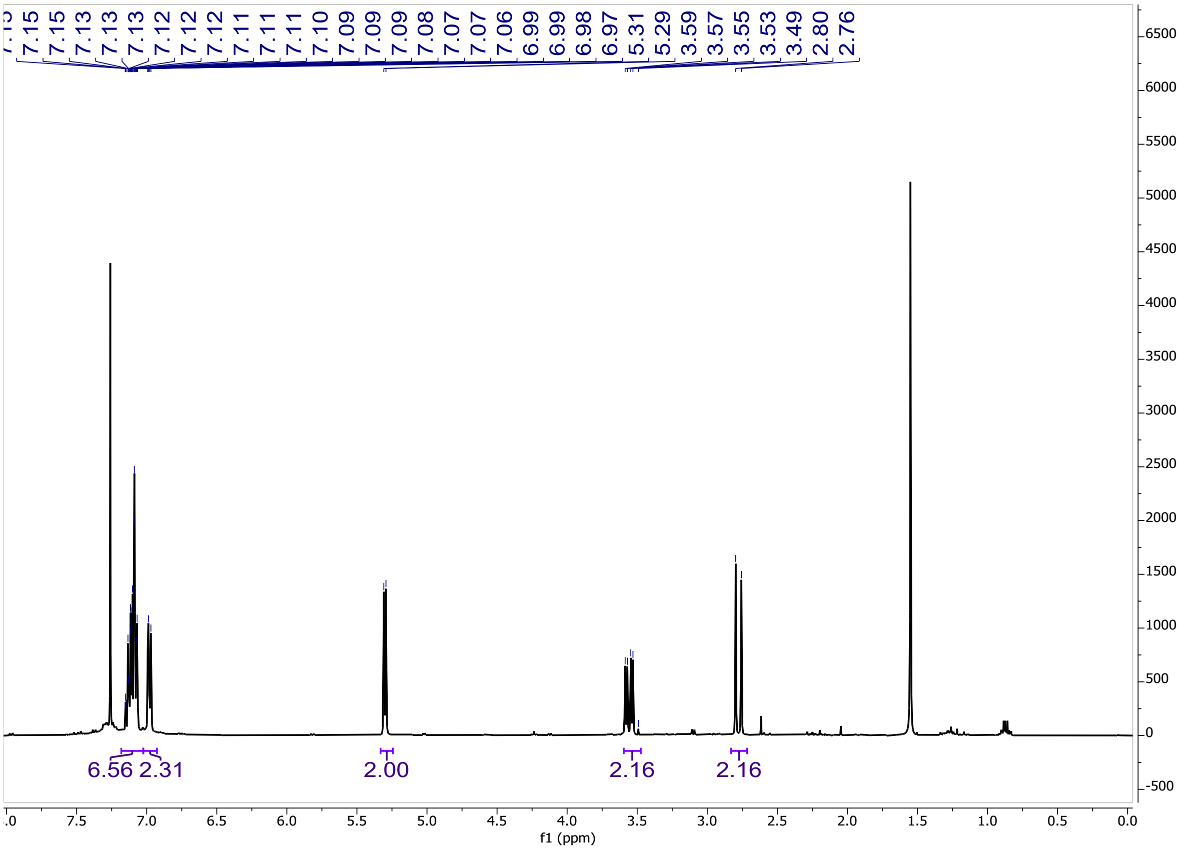

Figure S11. ^{1}H *NMR spectrum of intermediate* ***(1)***

Synthesis of intermediate (2)

To a solution of intermediate **(1)** (1 equiv, 14.17 mmol, 3.15 g) in dry THF (30 mL) was added n-butyllithium (1.6 M solution, 2 equiv, 28.34 mmol, 17.7 mL). The solution was stirred for 4 h at r.t., then quenched with water. THF was concentrated under reduced pressure, and the compound was extracted with DCM (5×), and washed with brine. The combined organic extracts were dried over $MgSO_4$, filtered, and concentrated under vacuum. The crude product was purified by column chromatography (silica gel, PE/EtOAc 5:1) to afford the intermediate **(2)** as a white solid (8.1 mmol, 1.8 g, 57%). The analytical data were in accordance with previously reported data.

^{1}H NMR (400 MHz, $CDCl_3$) δ 7.47 – 7.45 (m, 1H, Ar-*H*), 7.25 – 7.09 (m, 7H, 6 × Ar-*H*), 6.89 – 6.81 (m, 2H, R*H*C=CHR), 5.31-5.27 (dd, 1H, C*H*), 3.48-3.43 (dd, 1H, HC-*H*), 3.36-3.30 (dd, 1H, HC-*H*).

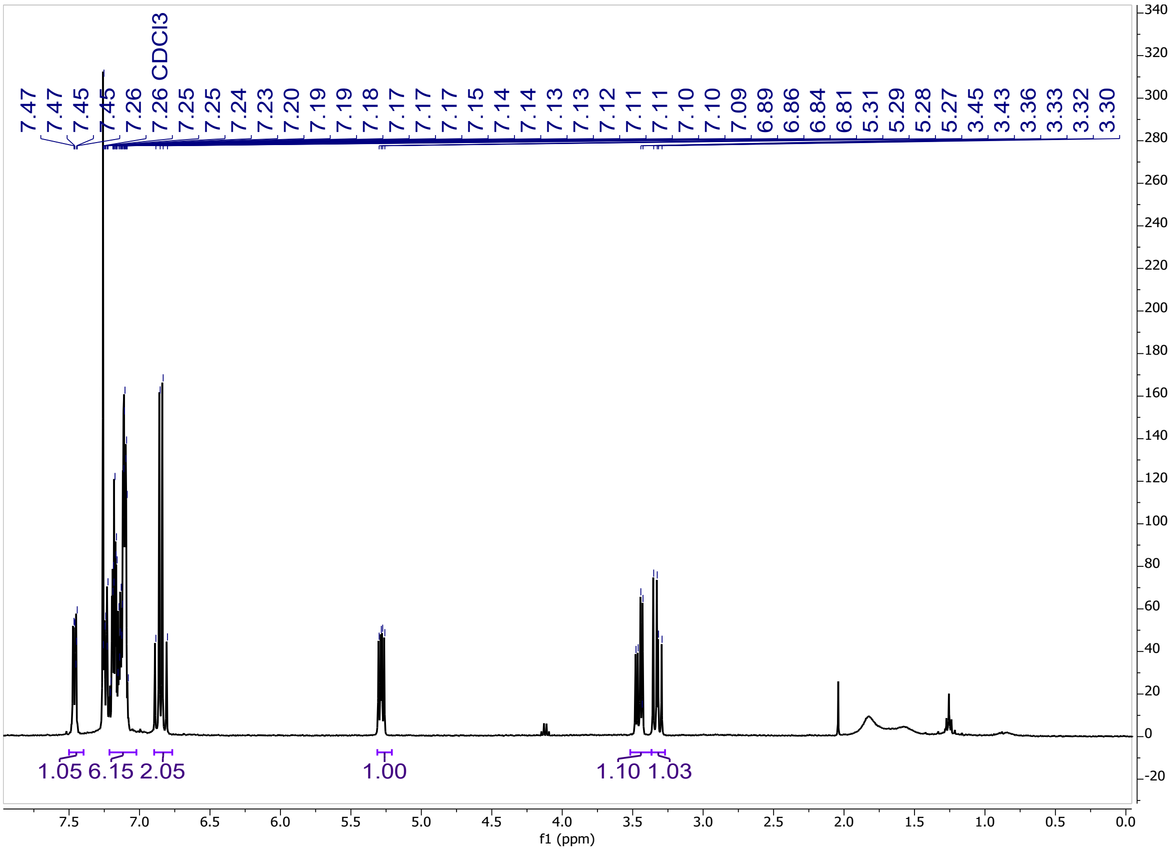

Figure S12. *^{1}H NMR spectrum of intermediate* ***(2)***

Synthesis of intermediate (3)

To a solution of intermediate **(2)** (1 equiv, 6.12 mmol, 1.36 g) in dry chloroform (40 mL) was added bromine (1 equiv, 6.12 mmol, 0.315 mL). The resulting mixture was stirred for 4 h at r.t. The solution was quenched with saturated $Na_2S_2O_3$ (40 mL) and the compound was extracted with chloroform. The organic phase was dried over $MgSO_4$, filtered, and concentrated under vacuum. The crude product was purified by column chromatography (silica gel, PE:DCM 2:1) to afford intermediate **(3)** as a white solid (2.7 mmol, 1.0 g, 44%). The analytical data were in accordance with previously reported data.

^{1}H NMR (400 MHz, $CDCl_3$) δ 7.69-7.67 (dt, 1H, Ar-*H*, diast. 1), 7.63 – 7.57 (m, 2H, 2 × Ar-*H*, diast. 2), 7.40 – 7.38 (d, 1H, Ar-*H*, diast. 1), 7.22 – 6.86 (m, 12H, 12 × Ar-*H*, diast. 1 and 2), 5.88 – 5.82 (m, 2H, Br-C*H*, diast 1), 5.76 (dd, 1H, C*H*, diast 2), 5.47-5.45 (dd, 1H, C*H*, diast 1), 5.33-5.28 (m, 2H, Br-C*H*, diast 2), 3.77-3.72 (ddd, 1H, HC-*H*, diast 1), 3.62-3.56 (dd, 1H, HC-*H*, diast 2), 3.12-3.07 (d, 1H, HC-*H*, diast 1), 2.87-2.83 (d, 1H, HC-*H*, diast 2).

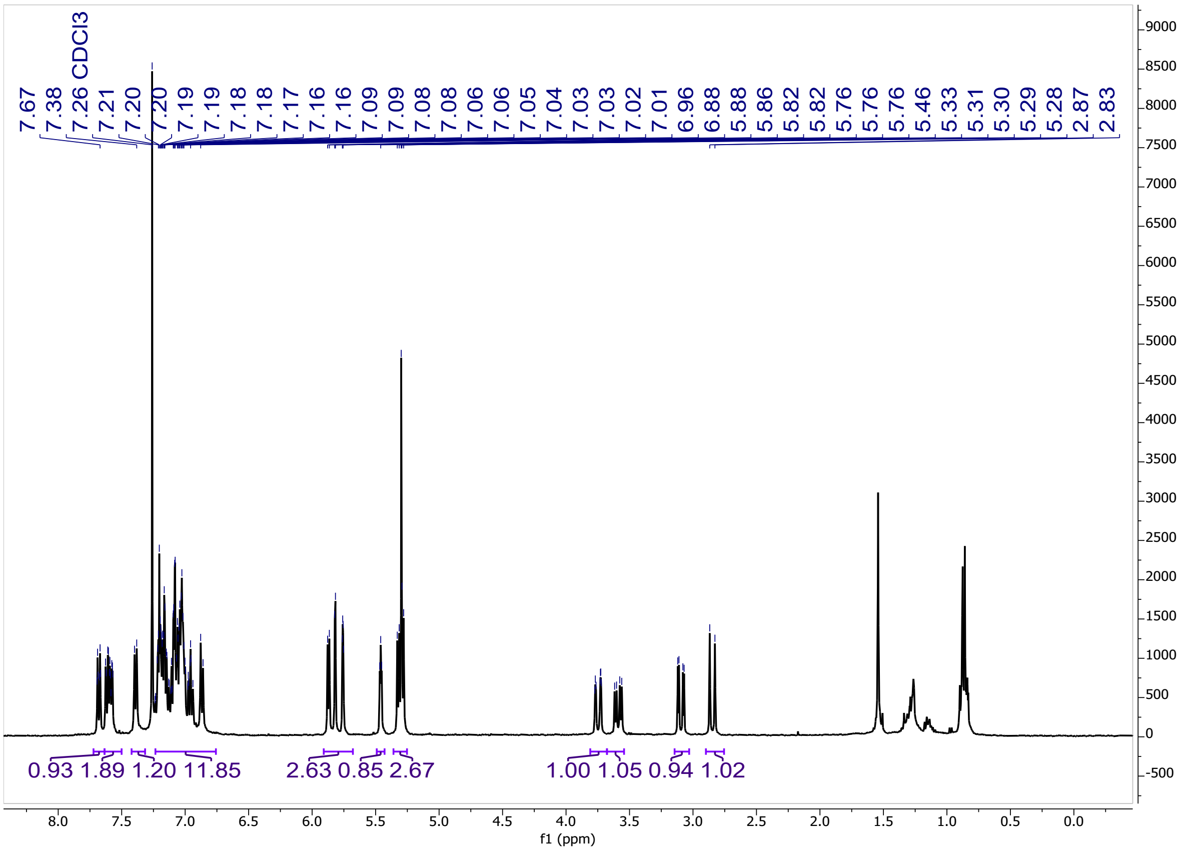

Figure S13. *^{1}H NMR spectrum of intermediate **(3)***

Synthesis of DIBO

Intermediate **(3)** (1 equiv, 3.16 mmol, 1.2 g) was dissolved in dry THF (20 mL) and the solution was cooled to 0°C. A fresh solution of lithium diisopropylamide (0.8 M in dry THF, 5.8 equiv, 18.32 mmol, 23.0 mL) was added and the resulting solution was stirred for 1 hour at r.t. The solution was quenched with water and THF was removed under reduced pressure. The product was extracted with DCM and washed with water (1×) and brine (1×). The crude product was purified via column chromatography (silica gel, PE:DCM 1:3) to afford **DIBO** as a white solid (2.0 mmol, 440 mg, 63%). The analytical data were in accordance with previously reported data.

^{1}H NMR (400 MHz, $CDCl_3$) δ 7.76-7.74 (dt, 1H, Ar-*H*), 7.45 – 7.28 (m, 7H, 7 × Ar-*H*), 4.65-4.63 (t, 1H, C*H*), 3.13-3.09 (dd, 1H, HC-*H*), 2.95-2.92 (dd, 1H, HC-*H*).

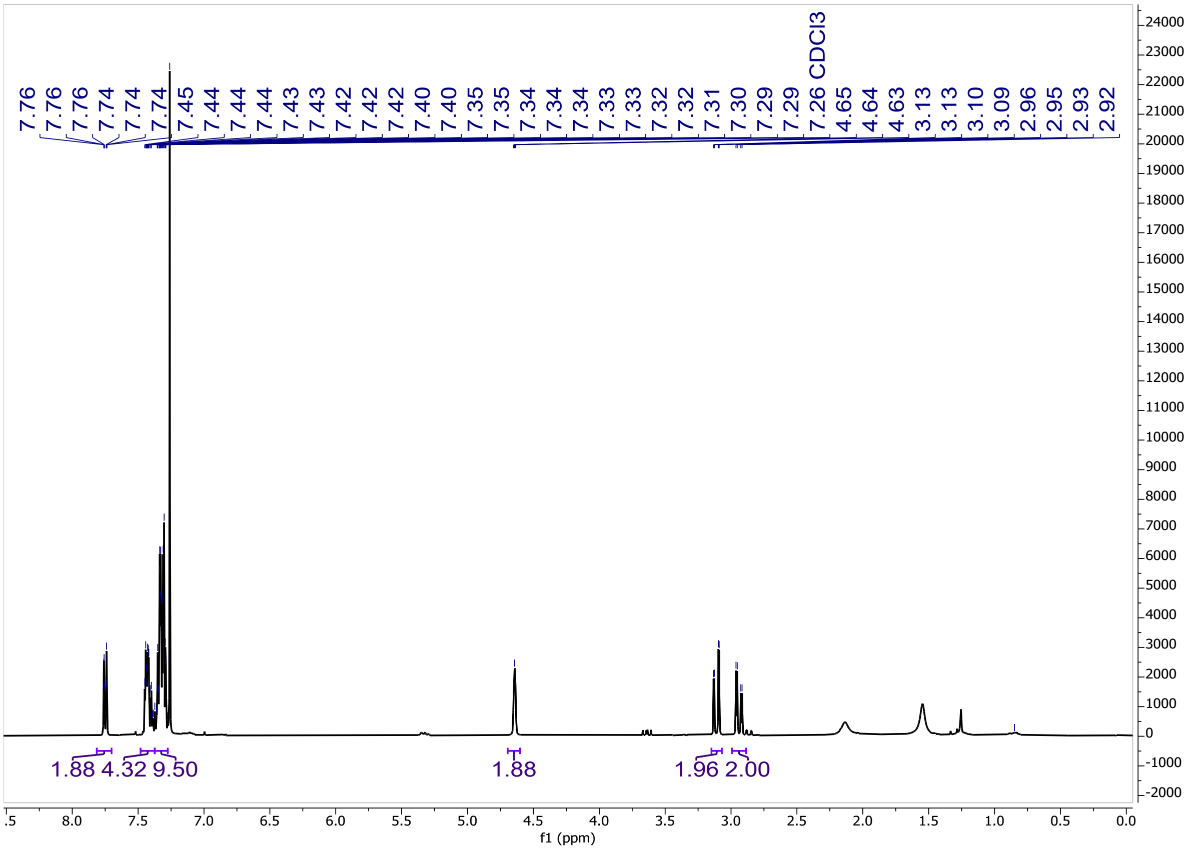

Figure S14. *^{1}H NMR spectrum of **DIBO***

Synthesis of DIBO-nitrophenyl

DIBO (1 equiv, 3.62 mmol, 796 mg) was dissolved in dry DCM (20 mL) under Ar. 4-nitrophenylchloroformate (2 equiv, 7.23 mmol, 1.46 g) and pyridine (5 equiv, 18.08 mmol, 1.45 mL) were added. The mixture was stirred for 16 h at r.t. The solution was concentrated under reduced pressure and the crude product was purified via column chromatography (silica gel, PE:DCM 1:1) to afford **DIBO-nitrophenyl** as a white solid (2.30 mmol, 884 mg, 63%). The analytical data were in accordance with previously reported data.

^{1}H NMR (400 MHz, $CDCl_3$) δ 8.30 – 8.27 (d, 2H, 2 × Ar-*H*), 7.63-7.61 (d, 1H, DIBO-*H*), 7.44 – 7.31 (m, 9H, 7 × DIBO-*H* and 2 × Ar-*H*), 5.59-5.58 (m, 1H, CH_{DIBO}), 3.36-3.32 (dd, 1H, HC-H_{DIBO}), 3.07-3.00 (dd, 1H, HC-H_{DIBO}).

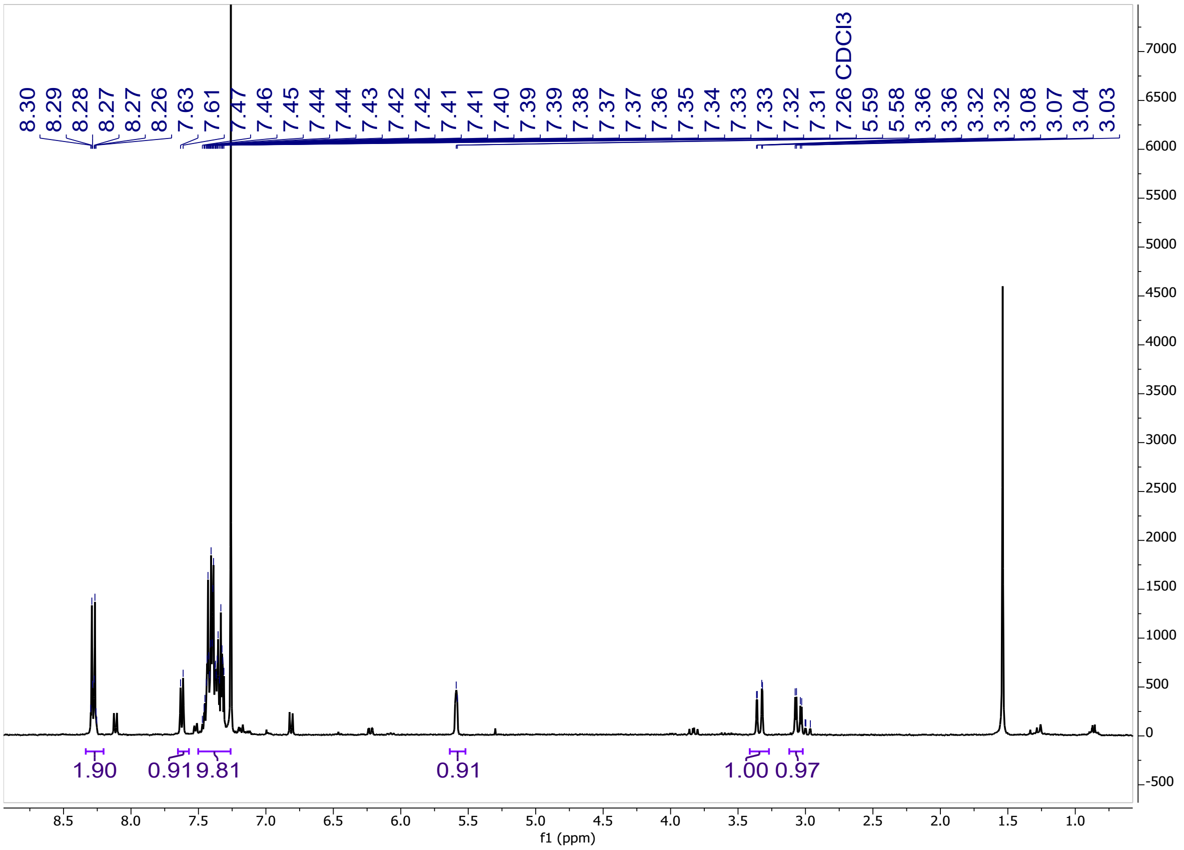

Figure S15. *^{1}H NMR spectrum of* ***DIBO-nitrophenyl***

Synthesis of APTES-DIBO

To a solution of DIBO-nitrophenyl (1 equiv, 0.16 mmol, 60 mg) in dry dioxane (7 mL) were added (3-aminopropyl)triethoxysilane (1.57 equiv, 0.24 mmol, 57 µL) and pyridine (3 equiv, 0.50 mmol, 40 µL). The resulting mixture was stirred overnight at r.t. The solution was concentrated under reduced pressure. The crude product was purified via column chromatography (silica gel, PE:EtOAc 8:2) to afford **APTES-DIBO** as a white solid (0.08 mmol, 38 mg, 47%).

^{1}H NMR (400 MHz, $CDCl_3$) δ 7.50-7.48 (d, 1H, 1 × Ar-H), 7.37-7.26 (m, 7H, 1 × Ar-H), 5.49 (s, 1H, CH_{DIBO}), 3.37-3.82 (q, 6H, CH_3-CH_2-O-Si), 3.24-3.13 (m, 3H, 2 × NH-CH_2-CH_2, 1 × dd, 1H, HC-*H*), 3.24-3.13 (dd, 1H, HC-*H*), 1.70-1.63 (q, 2H, CH_2-CH_2-NH), 1.29-1.23 (m, 9H, 3 × CH_3), 0.68-0.64 (t, 2H, Si-CH_2-CH_2).
HRMS (ESI/QTOF) m/z: $[M + Na]^+$ Calcd for $C_{26}H_{33}NNaO_5Si^+$ 490.2020; Found 490.2031

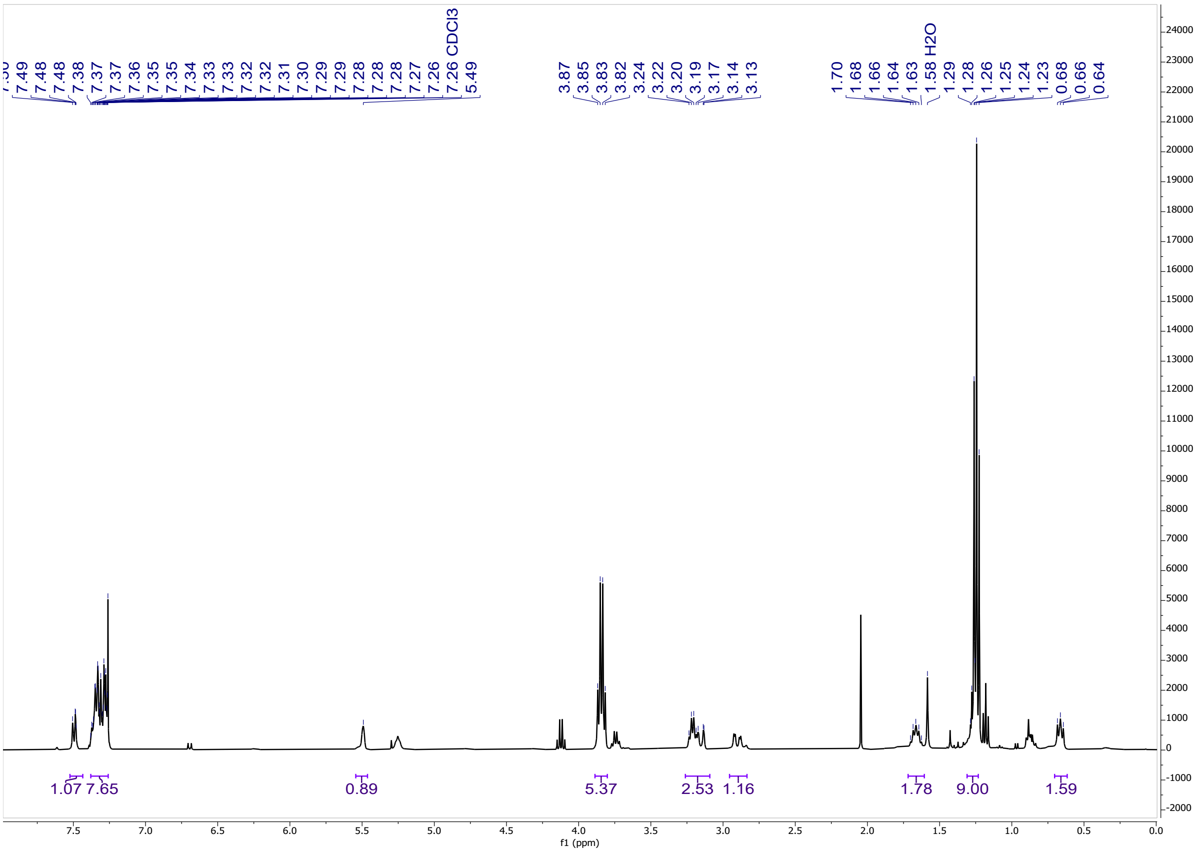

Figure S16. *^{1}H NMR spectrum of* ***APTES-DIBO***

2.3. Synthesis of fluorescent labelling reagents

2.3.1. Synthesis of N_3-cleaveable-FITC

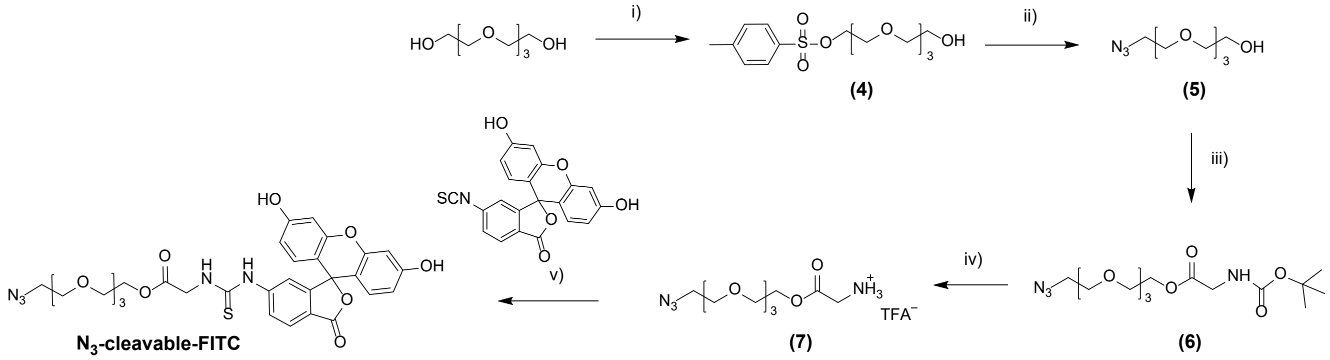

Scheme S4. *Synthesis of **N_3-cleavable-FITC***. Conditions: i) p-Toluenesulfonyl chloride, Et_3N, dry DCM, r.t, 2 days; ii) NaN_3, dry DMF, 90°C, 15h; iii) N-(tert-butoxycarbonyl)glycine, DMAP, DCC, r.t, overnight; iv) TFA, DCM, r.t, 5h ; v) FITC, Et_3N, dry THF, r.t, 3h.

Synthesis of intermediate (4)

Tetraethylene glycol (5 equiv, 514.9 mmol, 100.0 g) was dissolved in dry DCM (40 mL) under argon. The mixture was cooled to 0 °C. p-Toluenesulfonyl chloride (1.0 equiv, 103.0 mmol, 19.6 g) and triethylamine (Et_3N, 3 equiv, 308.91 mmol, 43.06 mL) were added. The mixture was stirred at r.t for 2 days. The reaction mixture was washed with water (3 × 50 mL). The organic phase was dried over $MgSO_4$, filtered and concentrated under vacuum. The crude product was purified by column chromatography (silica gel, DCM/MeOH 1:30) to afford the intermediate (4) as a colorless oil (76.72 mmol, 15.9 g, 44%). The analytical data were in accordance with previously reported data (K. Heller, P. Ochtrop, M. F. Albers, F. B. Zauner, A. Itzen and C. Hedberg, Angewandte Chemie International Edition, 2015, 54, 10327–10330).

^1H NMR (400 MHz, $CDCl_3$) δ 7.81-7.39 (d, J = 7.8 Hz, 2H, 2 × Ar-*H*), 7.35 – 7.33 (d, J = 7.3 Hz, 2H, 2 × Ar-*H*), 4.18 – 4.15 (m, 2H, C*H*$_2$-OTs), 3.72 – 3.59 (m, 14H, 3 × C*H*$_2$-O-C*H*$_2$ and CH_2-C*H*$_2$-OH), 2.45 (s, 3H, Ar-C*H*$_3$).

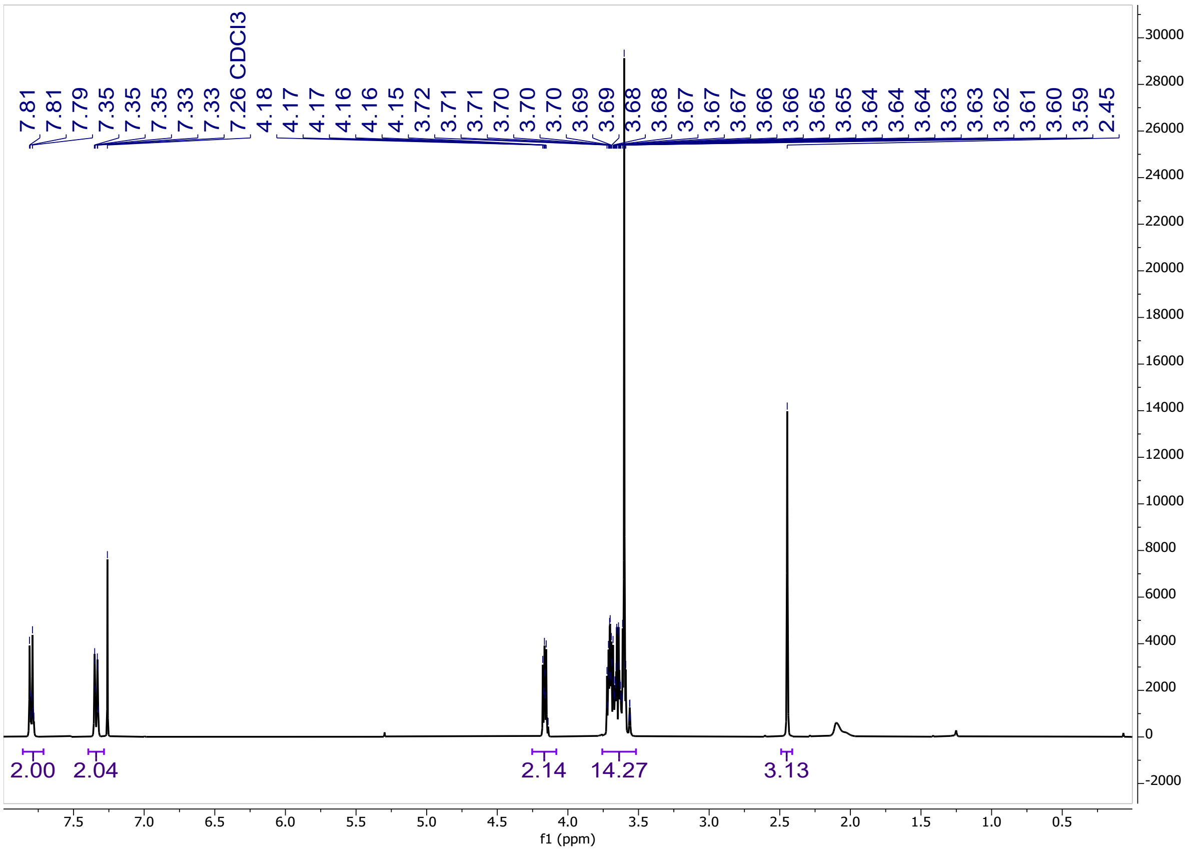

Figure S17. *^{1}H NMR spectrum of intermediate* ***(4)***

Synthesis of intermediate (5)

NaN_3 (1.2 equiv, 92.1 mmol, 6.0 g) was added to a solution of intermediate **(4)** (1.0 equiv, 76.7 mmol, 26.7 g) in dry DMF (25 mL). The resulting mixture was stirred at 90 °C for 15 h. The solvent was evaporated under vacuum and the product was extracted with EtOAc (30 mL) and washed with brine (3 × 30 mL). The organic layer was dried over $MgSO_4$ and concentrated in vacuo to afford intermediate **(5)** as a yellow oil (24.2 mmol, 5.3 g, 29%). The analytical data were in accordance with previously reported data (S. M. F. M. Passemard, EPFL, 2014).

^{1}H NMR (400 MHz, $CDCl_3$) δ 3.74 – 3.72 (m, 2H, CH_2-OH), 3.69 – 3.67 (m, 10H, 5 × CH_2-O-CH_2), 3.63 – 3.61 (m, 2H, CH_2-CH_2-N_3), 3.41-3.38 (td, 2H, CH_2-N_3).

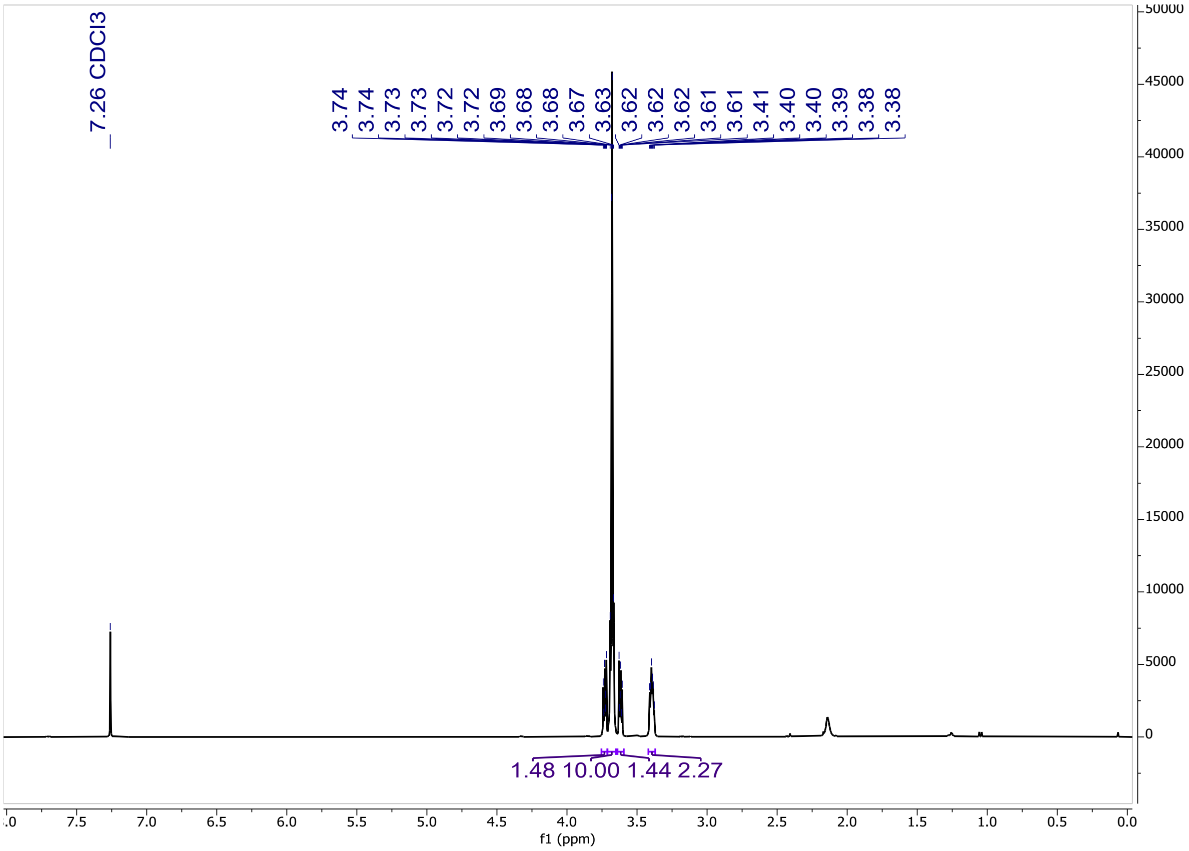

Figure S18. *^{1}H NMR spectrum of intermediate* ***(5)***

Synthesis of intermediate (6)

To a solution of intermediate **(5)** (1 equiv, 0.48 mmol, 105 mg) in dry DCM (8 mL) were added N-(tert-butoxycarbonyl)glycine (0.95 equiv, 0.45 mmol, 105 mg), 4-(dimethylamino)pyridine (DMAP, 1.05 equiv, 0.50 mmol, 56 mg) and dicyclohexyl carbodiimide (DCC, 1.05 equiv, 0.50 mmol, 103.5 mg). The resulting mixture turned blurry after 5 minutes and was stirred overnight at r.t. The solvent was removed under vacuum, and the product was extracted with DCM and washed with water. The organic phase was dried over $MgSO_4$, filtered and concentrated under vacuum. The crude product was purified by column chromatography (silica gel, DCM/MeOH 100:0 to 95:5) to afford intermediate **(6)** as a white solid (0.33 mmol, 122.4 mg, 72%).

^{1}H NMR (400 MHz, $CDCl_3$) δ 4.30 – 4.28 (t, 2H, CH_2-O-C=O), 3.93-3.92 (d, 2H, CH_2-C=O-O), 3.71-3.64 (m, 12H, 6 × CH_2-O), 3.38 – 3.36 (t, 2H, CH_2-N_3), 1.43 (s, 9H, CH_3). HRMS (ESI/QTOF) m/z: [M + Na]$^+$ Calcd for $C_{15}H_{28}N_4NaO_7^+$ 399.1850; Found 399.1842

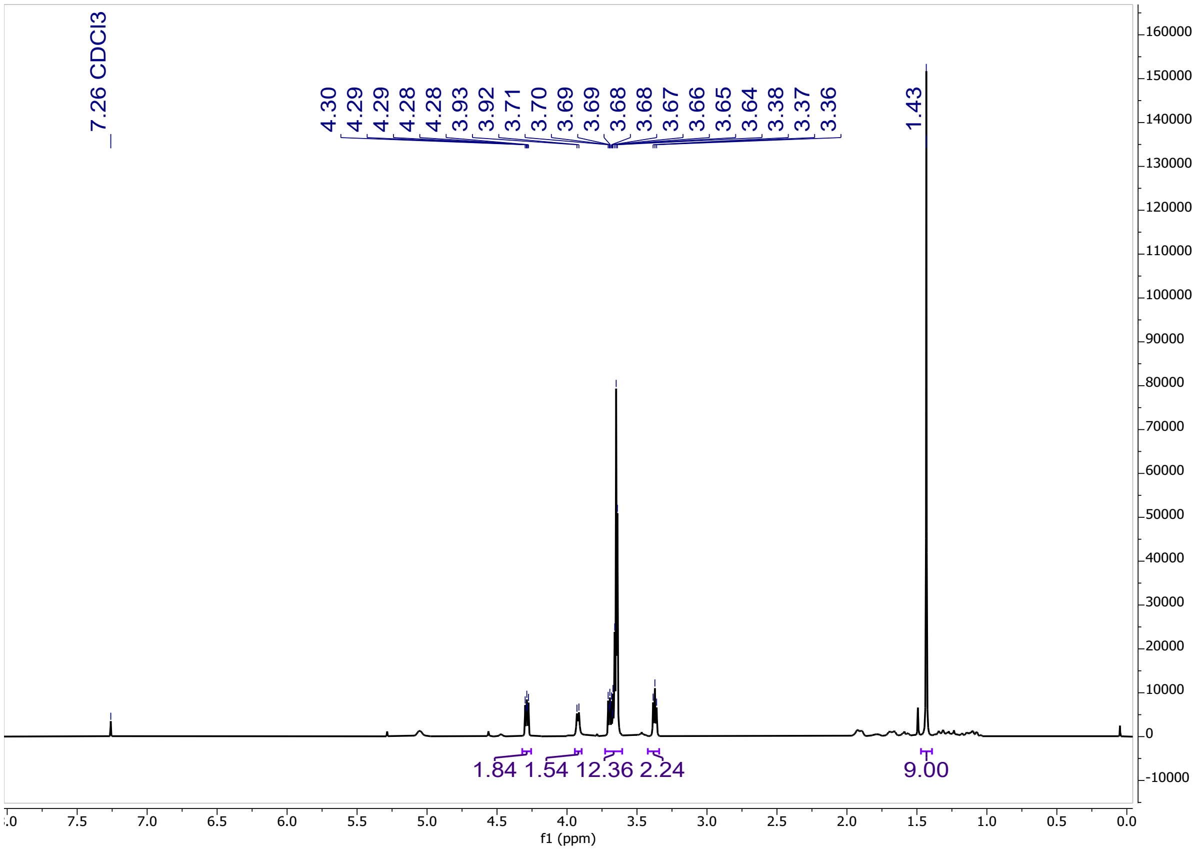

Figure S19.*^{1}H NMR spectrum of intermediate* ***(6)***

Synthesis of N_3-cleavable-FITC

To a solution of intermediate **(6)** (0.096 mmol, 36.2 mg) in dry DCM (5 mL), trifluoroacetic acid was added (TFA, 1mL). The mixture was stirred for 5 h at r.t. The solvent was removed under air flow. The resulting intermediate **(7)** (HRMS (ESI/QTOF) m/z: $[M + H]^+$ Calcd for $C_{10}H_{21}N_4O_5^+$ 277.1506; Found 277.1503) was used without any further purification.

To a solution of intermediate **(7)** (1 equiv, 0.58 mmol, 225 mg) in dry THF (10 mL) under argon (brown glassware), Et_3N (3.5 equiv, 2.02 mmol, 281 µL) and fluorescein isothiocyanate (FITC, 0.5 equiv, 0.29 mmol, 112 mg) were added. The resulting mixture was stirred for 3 h at r.t. The solvent was evaporated under reduced pressure and the crude product was purified via reversed-phase chromatography (MeOH:H_2O 1:2 + TFA 0.1% to MeOH:H_2O 1:1 + TFA 0.1%) to afford **N_3-cleavable-FITC** as an orange solid (0.03 mmol, 23 mg, 11%).

^{1}H NMR (400 MHz, MeOD) δ 8.33-8.31 (m, 1H, C*H*-Ar), 7.90-7.87 (dd, 1H, C*H*-Ar), 7.23-7.18 (d, 1H, CH-Ar), 6.98-6.91 (d, 2H, 2×C*H*-Ar), 6.84-6.83 (d, 2H, 2×C*H*-Ar), 6.72-6.67 (dd, 2H, 2×C*H*-Ar), 4.41 (s, 2H, C*H*$_2$-NH-C=S), 4.31-4.29 (d, 2H, CH$_2$-C*H*$_2$-O-C=O), 3.74-3.71 (t, 2H, C*H*$_2$-CH$_2$-O-C=O), 3.66-3.51 (m, 10H, 5×C*H*$_2$-O), 3.35-3.30 (m, C*H*$_2$-N$_3$ and MeOD solvent residual peak). HRMS (ESI/QTOF) m/z: $[M + H]^+$ Calcd for $C_{31}H_{32}N_5O_{10}S^+$ 666.1864; Found 666.1870.

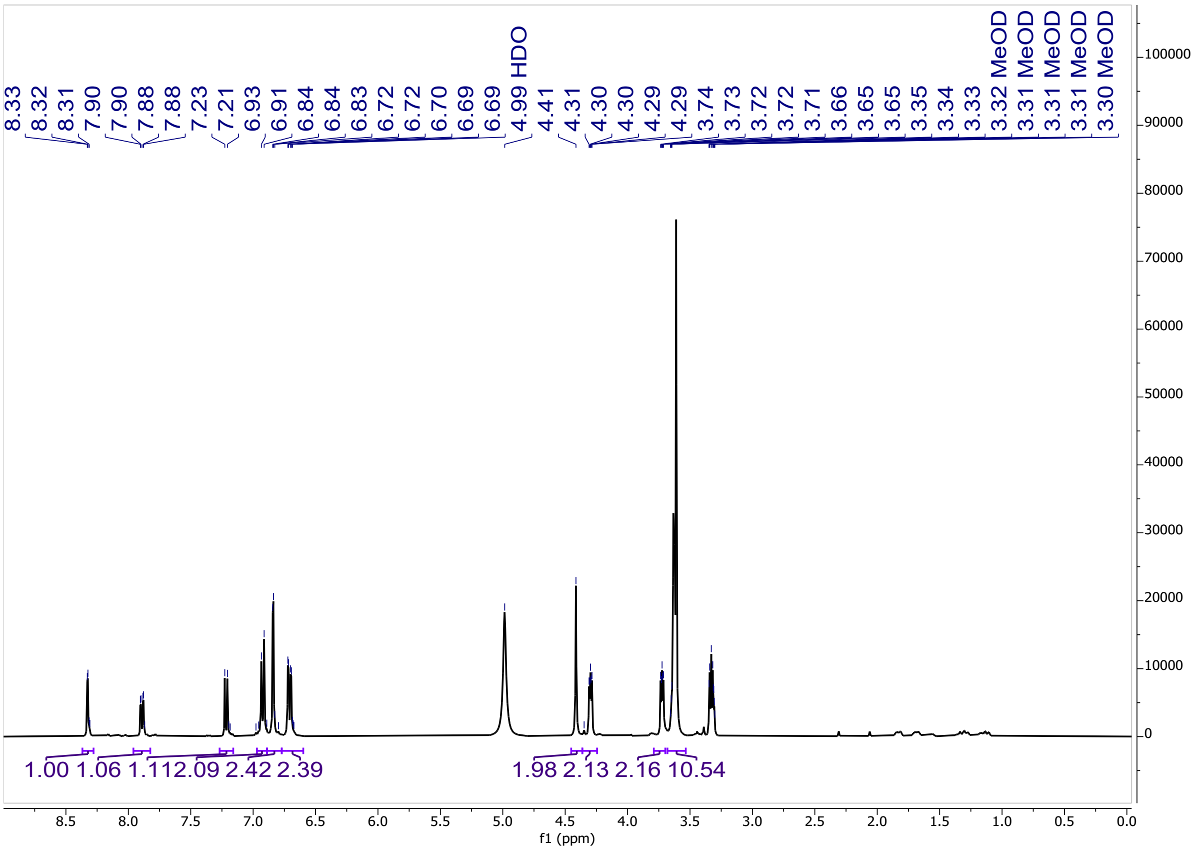

Figure S20. *^{1}H NMR spectrum of **N₃-cleaveable-FITC***

2.3.2. Synthesis of alkyne-cleavable-FITC

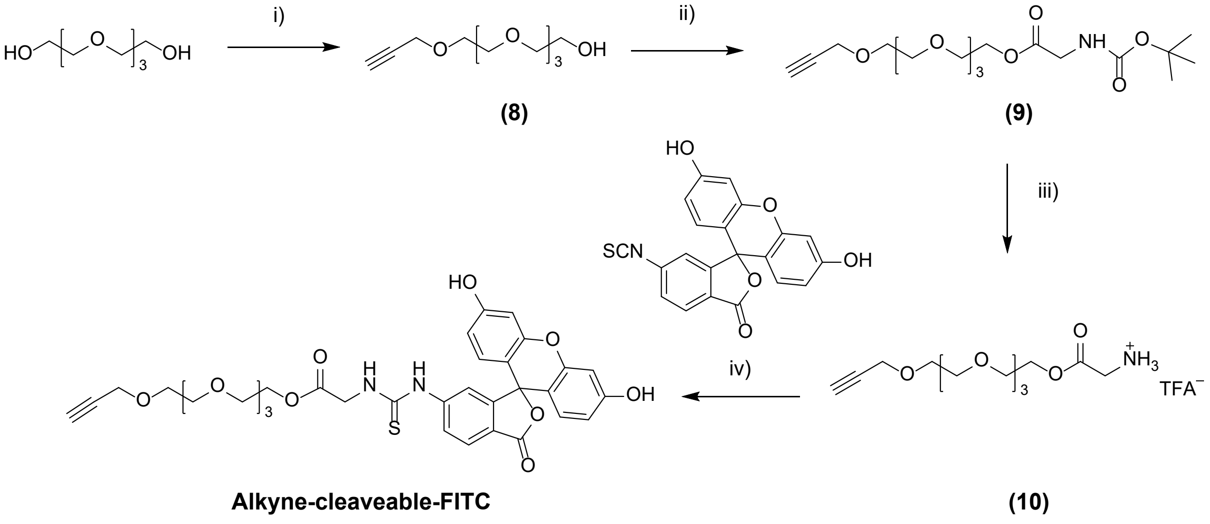

Scheme S5. *Synthesis of* ***alkyne-cleavable-FITC***. Conditions: i) NaH, tetraethylene gycol, propargyl bromide, r.t, overnight; ii) N-(tert-butoxycarbonyl)glycine, DMAP, DCC, r.t, overnight; iv) TFA, DCM, r.t, overnight ; v) FITC, Et_3N, dry THF, r.t, 6h.

Synthesis of intermediate (8)

To a solution of NaH (60%, 0.7 equiv, 1.85 mmol) in dry THF (7 mL) under argon, tetraethylene glycol (1 equiv, 2.57 mmol, 444 µL) was added. Then, propargyl bromide was added and the mixture was stirred at r.t overnight. The reaction mixture was quenched with water and extracted with DCM. The organic phase was dried over $MgSO_4$, filtered, and concentrated under vacuum. The crude product was

purified by column chromatography (silica gel, DCM:MeOH 100:0 to 97:3) to afford intermediate **(8)** as yellow oil (1.69 mmol, 393 mg, 91%).
The analytical data were in accordance with previously reported data (L. N. Goswami, Z. H. Houston, S. J. Sarma, S. S. Jalisatgi and M. F. Hawthorne, *Org Biomol Chem*, 2013, **11**, 1116–1126).

^{1}H NMR (400 MHz, $CDCl_3$) δ 4.09 (d, 2H, C*H*$_2$-C≡CH), 3.61-3.55 (m, 14H, C*H*$_2$-O), 3.50-3.48 (m, 2H, C*H*$_2$-OH), 2.39-2.37 (t, 1H, C*H*≡C).

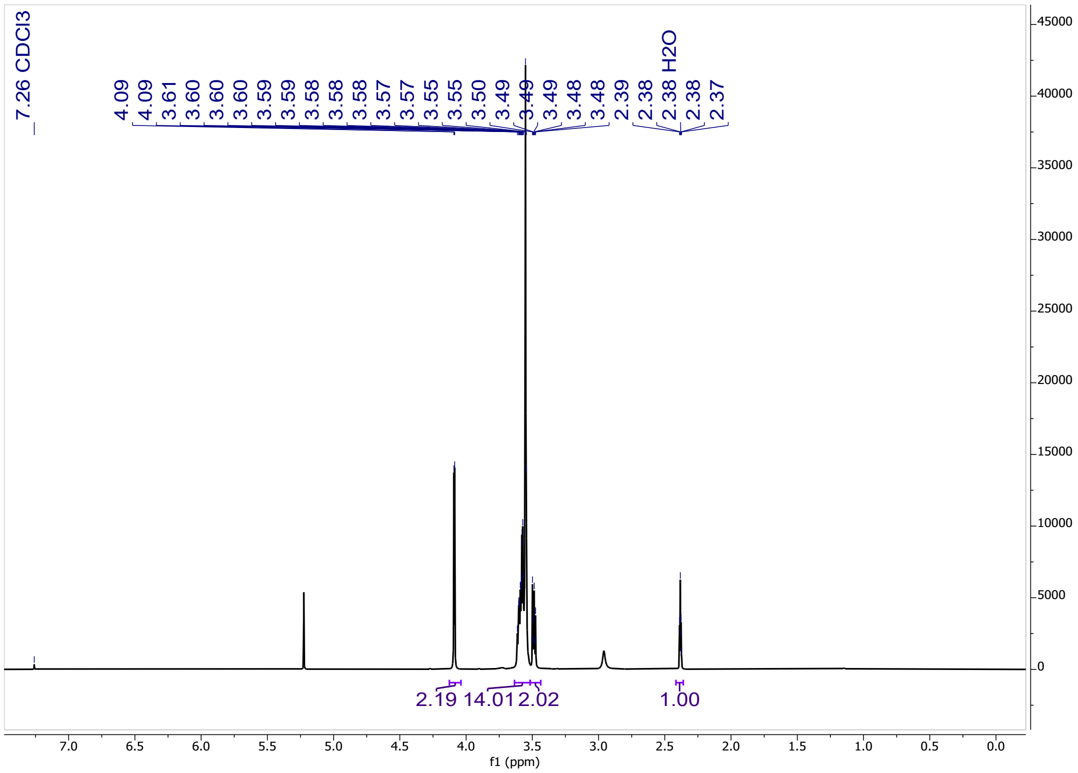

Figure S21. *^{1}H NMR spectrum of intermediate **(8)***

Synthesis of intermediate (9)

To a solution of intermediate **(8)** (1 equiv, 0.48 mmol, 105 mg) in dry DCM (15 mL), were added N-(tert-butoxycarbonyl)glycine (0.95 equiv, 0.45 mmol, 105 mg), DMAP (1.05 equiv, 0.50 mmol, 56 mg) and dicyclohexyl carbodiimide (1.05 equiv, 0.50 mmol, 103.5 mg). The resulting mixture turned blurry after 5 minutes and was stirred overnight at r.t. The solvent was removed under vacuum, and the product was extracted with DCM and washed with water. The organic phase was dried over $MgSO_4$, filtered, and concentrated under vacuum. The crude product was purified by column chromatography (silica gel, DCM/MeOH 100:0 to 95:5) to afford intermediate **(9)** as a white solid (0.33 mmol, 122.4 mg, 72%).

^{1}H NMR (400 MHz, $CDCl_3$) δ 4.31-4.29 (t, 2H, C*H*$_2$-O-C=O), 4.20 (d, 2H, C*H*$_2$-C≡CH), 3.94-3.93 (d, 2H, C*H*-NH-C=O), 3.72-3.65 (m, 14H, C*H*$_2$-O), 2.43 (t, 1H, C*H*≡C).
HRMS (ESI/QTOF) m/z: [M + Na]+ Calcd for $C_{18}H_{31}NNaO_8$+ 412.1942; Found 412.1954.

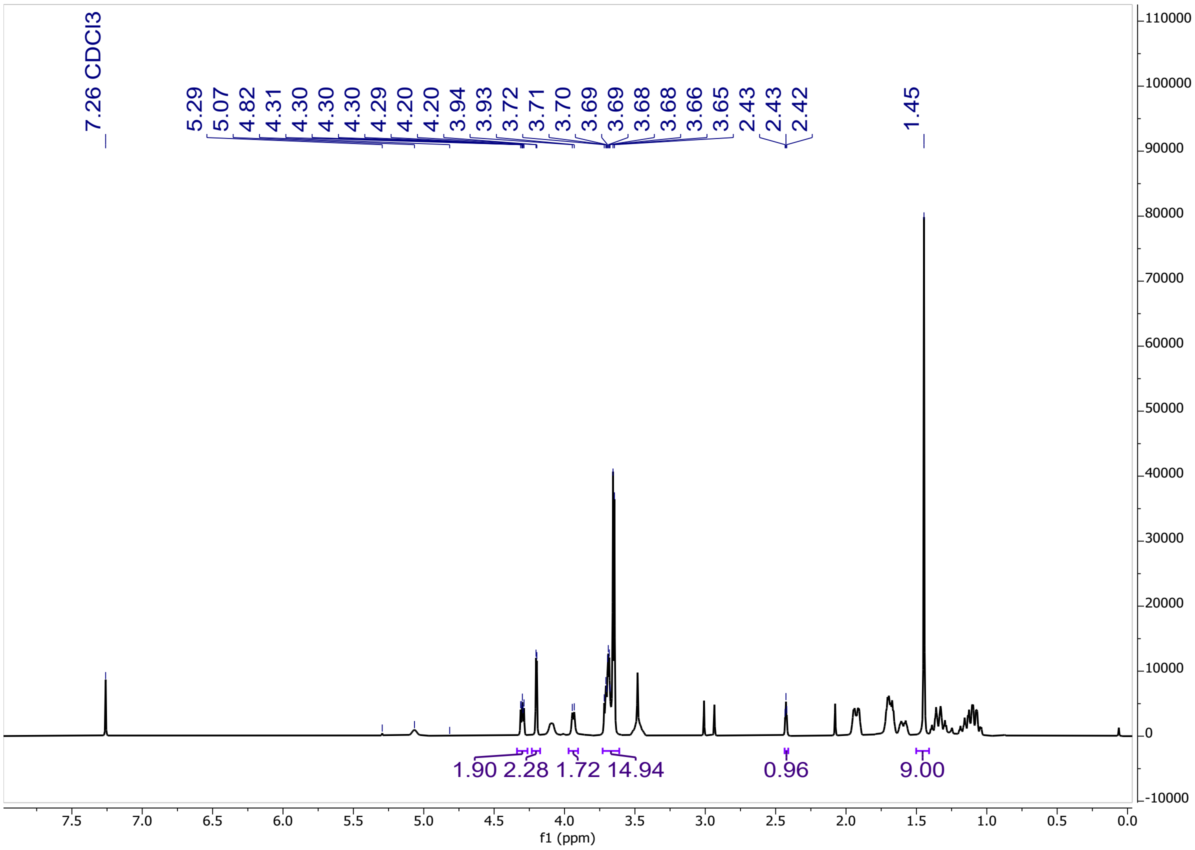

Figure S22. *^{1}H NMR spectrum of intermediate **(9)***

Synthesis of alkyne-cleavable-FITC

To a solution of intermediate **(9)** (1 equiv, 0.126 mmol, 49 mg) in dry DCM (10 mL), TFA (1 mL) was added under Ar. The resulting mixture was stirred overnight at r.t. The volatiles were removed under air flow and the resulting intermediate **(10)** (HRMS (ESI/QTOF) m/z: [M]+ Calcd for $C_{13}H_{24}NO_6$+ 290.1598; Found 290.1604) was used without further purification.

To a solution of intermediate **(10)** (1 equiv, 0.248 mmol, 100 mg) in dry THF (10 mL), were added Et_3N (3.5 equiv, 1.79 mmol, 250 µL) and FITC (1 equiv, 0.246 mmol, 96 mg) under argon in brown glassware. The mixture was stirred for 6 h at r.t., and the solvent was evaporated under reduced pressure. The crude product was purified by reversed-phase chromatography (MeOH:H_2O 1:2 + TFA 0.1% to MeOH:H_2O 7:3 + TFA 0.1%) to afford **alkyne-cleavable-FITC** as an orange solid (0.04 mmol, 27 mg, 16%).

^{1}H NMR (400 MHz, MeOD): δ 8.27 (m, 1H, C*H*-Ar), 7.90-7.88 (d, 1H, C*H*-Ar), 7.24-7.22 (d, 1H, C*H*-Ar), 6.89-6.87 (d, 2H, 2×C*H*-Ar), 6.83-6.82 (d, 2H, 2×C*H*-Ar), 6.70-6.68 (dd, 2H, 2×C*H*-Ar), 4.43 (s, 2H, C*H*$_2$-NH-C=S), 4.33-4.31 (t, 2H, CH$_2$-C*H*$_2$-O-C=O), 4.17-4.16 (d, 2H, CH$_2$-C≡CH), 3.76-3.74 (m, 2H, C*H*$_2$-CH$_2$-O-C=O), 3.68-3.62 (m, 12H, 3×C*H*$_2$-CH$_2$-O, 3×CH$_2$-C*H*$_2$-O), 2.83-2.81 (t, 1H, C≡C-*H*)**.** HRMS (ESI/QTOF) m/z: $[M + H]^+$ Calcd for $C_{34}H_{35}N_2O_{11}S^+$ 679.1956; Found 679.1967.

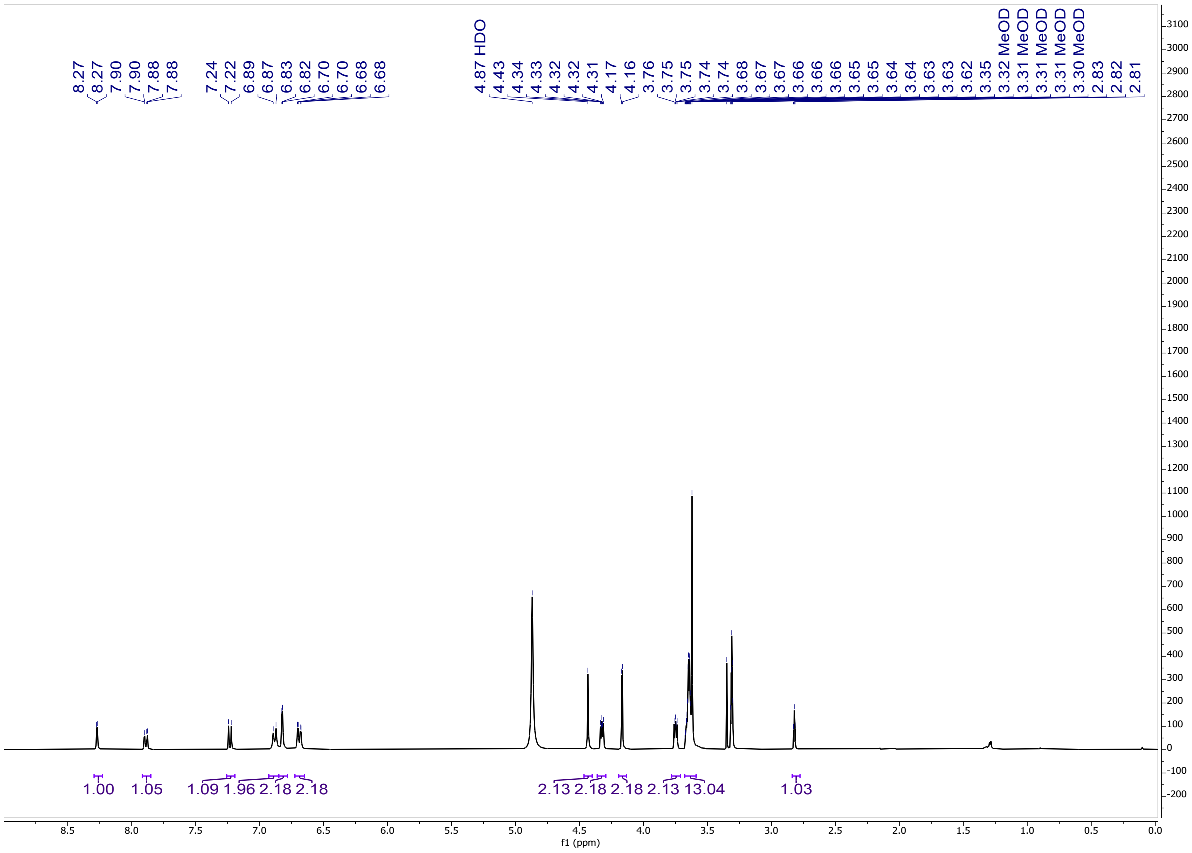

Figure S23. ^1H *NMR spectrum of* ***alkyne-cleavable-FITC***

3. Surface characterization

3.1. Quantification of surface reactive groups after silanization

The quantification protocol was adapted from the procedure previously reported by Miyahara, K. et al. (1 L. N. Goswami, Z. H. Houston, S. J. Sarma, S. S. Jalisatgi and M. F. Hawthorne, *Org Biomol Chem*, 2013, **11**, 1116–1126.

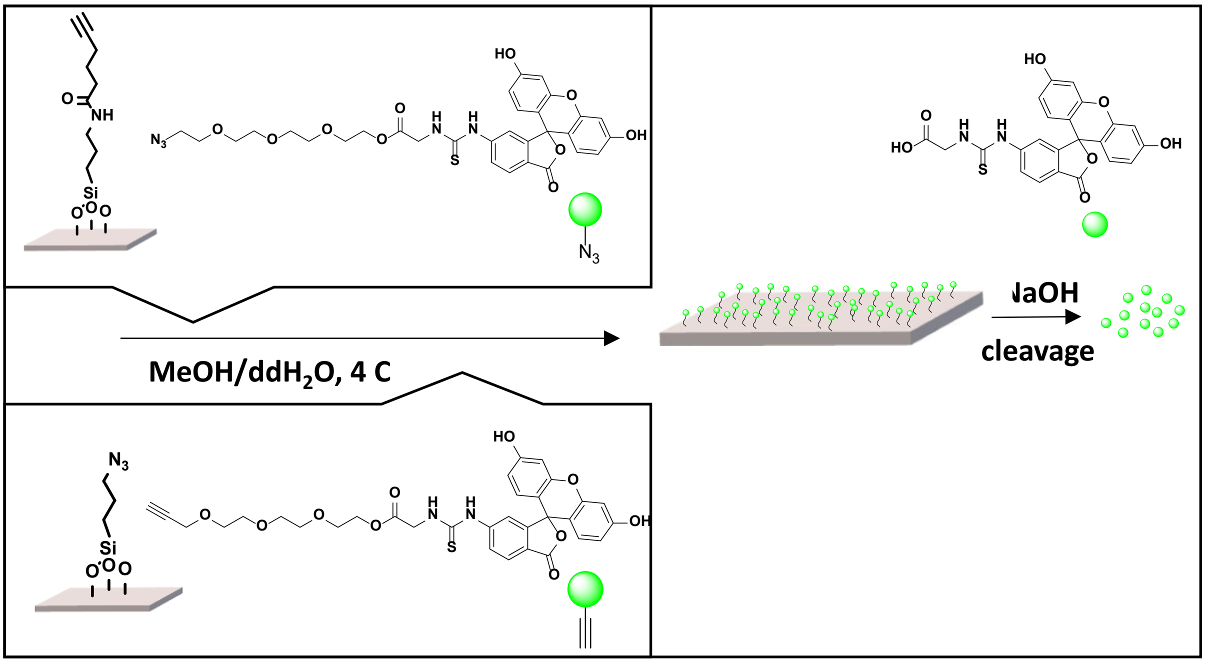

Figure S24. *Schematic representation of the quantification of surface azide and alkyne reactive groups.*

3.2. XPS analysis of functionalized slides

3.2.1. Characterization of silanized surfaces

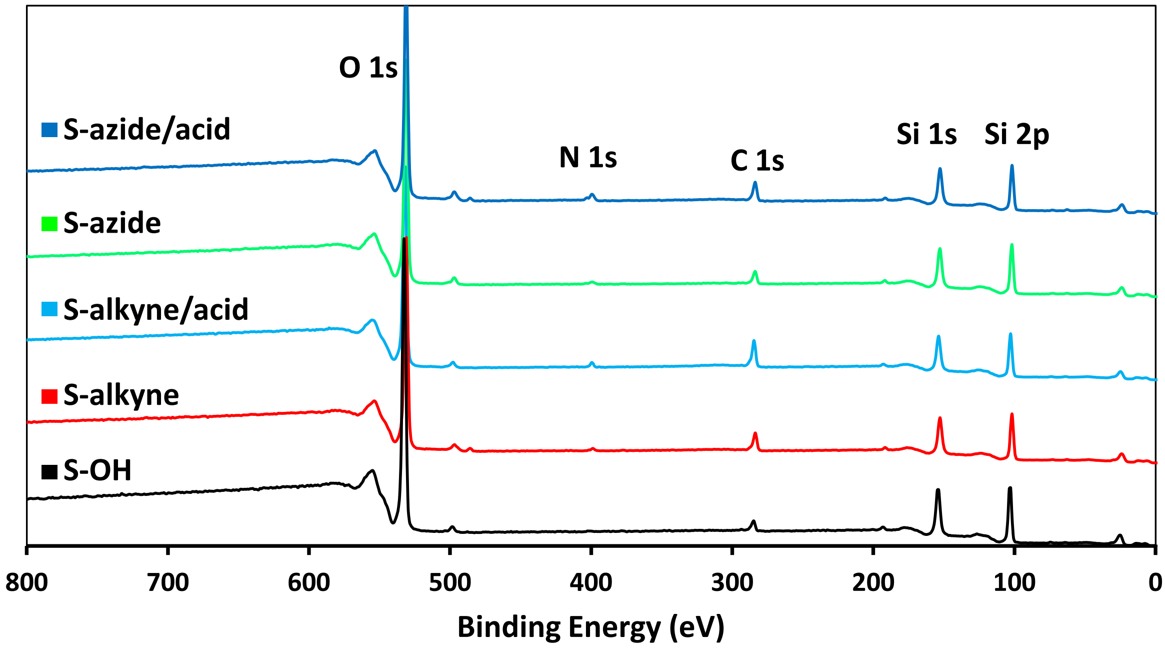

Figure S25. *XPS survey spectra of **S-OH, S-azide** and **S-alkyne** slides*. Comparison of silanized slides prepared with or without the addition of valeric acid.

Table S1. *Surface relative atomic concentration of C, N, O and Si detected via XPS for silanized surfaces*. Silanization was performed with or without the addition of valeric acid. Surface compositions are expressed as atomic percentage (%).

Slides	C 1s	N 1s	O1s	Si 2p	C/N	C/Si	N/Si
**S-alkyne**	13.9	0.8	57.2	28.1	17.0	0.49	0.03
**S-alkyne**/acid	20.5	1.6	51.0	26.9	13.0	0.76	0.06
**S-azide**	10.5	0.8	58.8	30.0	12.9	0.35	0.03
**S-azide**/acid	16.0	2.0	54.3	27.7	8.0	0.58	0.07
**S-DIBO**/acid	16.5	0.6	57.4	25.4	25.6	0.65	0.03

3.2.2. Characterization of peptide conjugated surfaces

Table S2. *Surface relative atomic concentration of C, N, O and Si detected via XPS for peptide-conjugated surfaces*. Surface compositions are expressed as atomic percentage (%).

Slides	C 1s	N 1s	O1s	Si 2p	C/N
**S-alkyne-P**	30.9	6.5	41.8	20.8	4.8
**S-azide-P**	21.5	3.5	51.8	23.2	6.1
**S-DIBO-P**	16.0	0.7	57.4	26.0	24.3

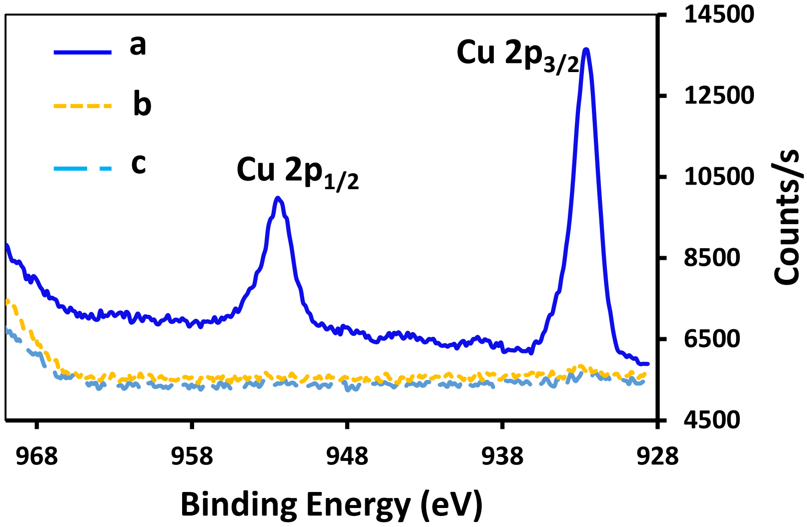

Figure S26. *High-resolution XPS spectra of Cu 2p region on **S-alkyne-P** slides*. a) washing slides with miliQ-water and acetonitrile; b) washing slide with 0.1% Tween-20; c) slides treated with Cyclam (2 mg.mL^{-1}).

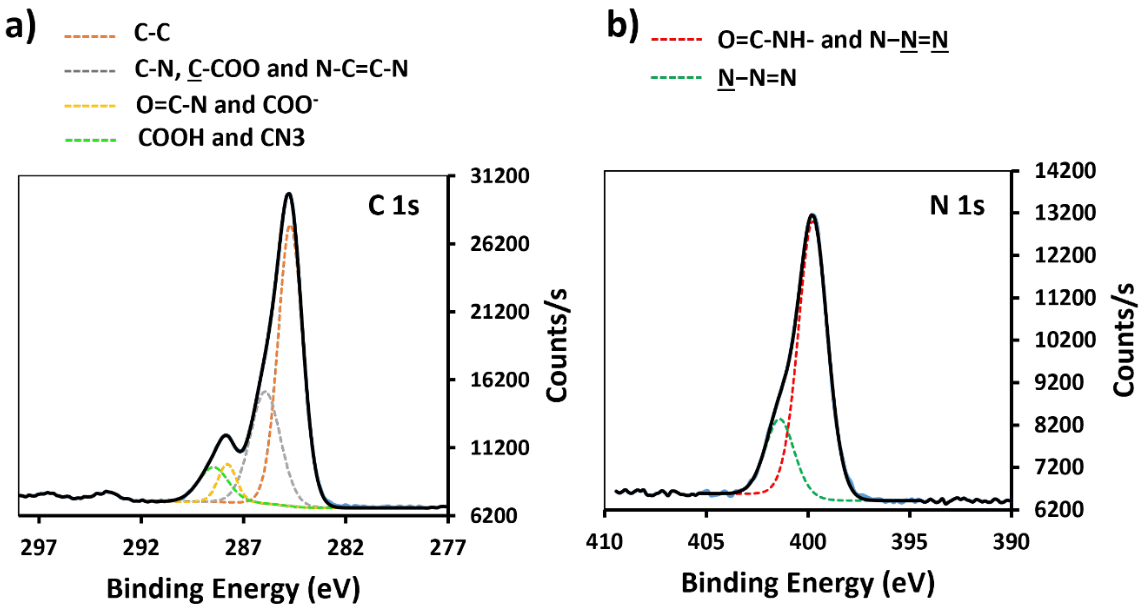

Figure S27. *High resolution XPS data of **S-azide-P** slides*. a) C 1s XPS spectrum; b) N 1s spectrum.

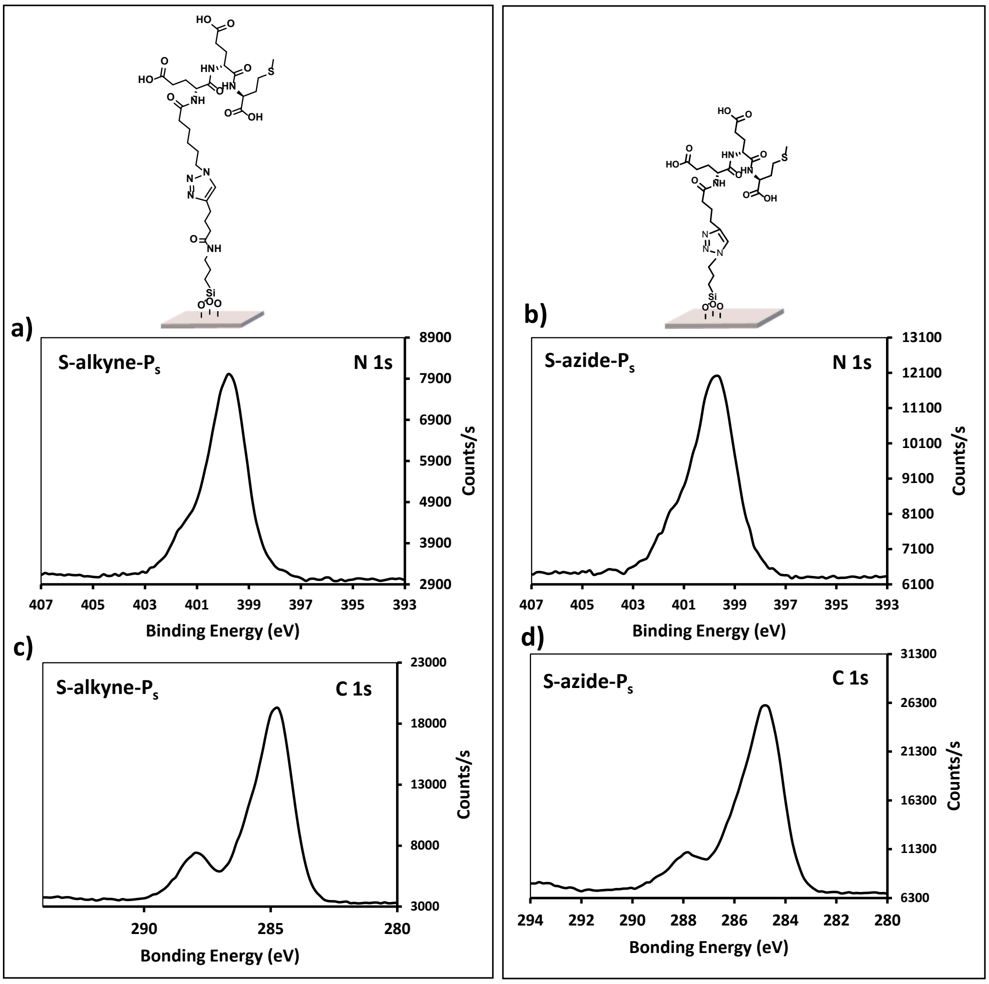

Figure S28. *High resolution XPS spectra of* ***S-alkyne-Ps*** *and* ***S-azide-Ps*** *slides*. a), b) N 1s spectra; c), d) C 1s spectra. Reaction conditions for peptide conjugation: 3.6 mM of peptide, $CuSO_4$, THPTA, H_2O/MeOH 1:1, 4h, 25°C.

3.2.3. Characterization of DNA functionalized surfaces

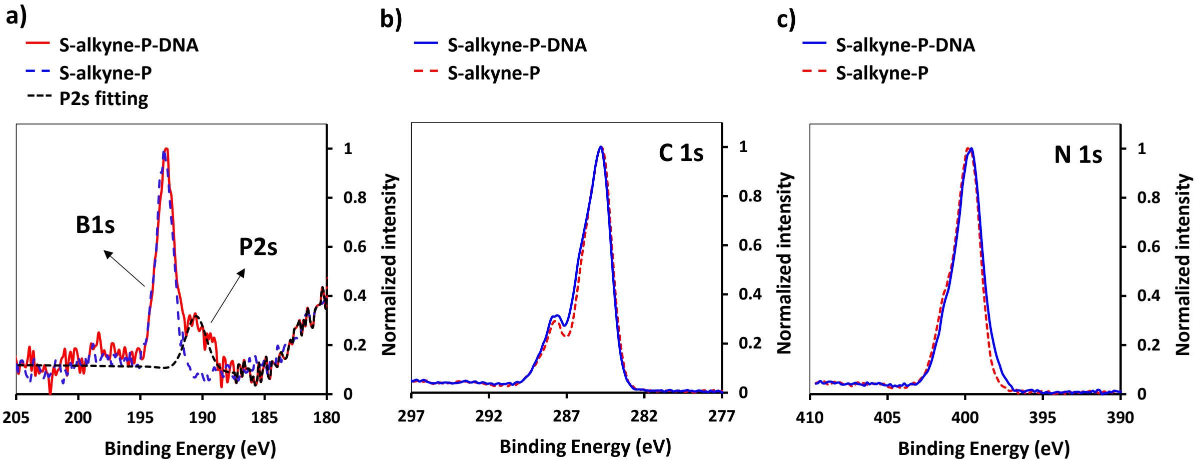

Figure S29. *High resolution XPS data of DNA functionalized slides*. a) P 2s XPS spectra of **S-alkyne-P** and **S-alkyne-P-DNA***;* b) C 1s XPS spectra of **S-alkyne-P** and **S-alkyne-P-DNA**; c) N 1s spectra of **S-alkyne-P** and **S-alkyne-P-DNA**.

Table S3. *Surface relative atomic concentration of C, N, O, P and Si detected via XPS for DNA functionalized slides.* Surface compositions are expressed as atomic percentage (%).

**Slides**	C 1s	N 1s	O1s	P 2p	Si 2p
**S-alkyne-P-DNA**	37.0	8.8	36.1	0.46	17.6
**S-azide-P-DNA**	28.5	7.1	42.5	0.24	21.7
**S-DIBO-P-DNA**	23.0	3.2	51.0	0.16	22.7

3.3. Stability of DNA functionalized slides upon storage at 4°C

In order to assess the stability of the sensing surfaces, DNA functionalized slides were immersed in 4 mL of MilliQ water and kept at 4 °C for 4 weeks. Quantification of the hybridization density was performed on the freshly functionalized slides and after 4 weeks of storage.

Table S4. *Hybridization density measured at the surface of DNA functionalized slides*. Results are expressed as mean values ± SD (n independent experiments).

**Slides**	hybridization density (pmol.cm^{-2})[a]	hybridization density (pmol.cm^{-2})[b]
**S-alkyne-P-DNA**	2.9 ± 0.8 (n=3)	2.0 ± 0.2 (n = 3)
**S-DIBO-P-DNA**	2.32 ± 0.18 (n=4)	2.5 ± 0.9 (n = 3)

[a]Quantification was performed on freshly functionalized slides. [b]Quantification was performed after storage of the slides for 4 weeks at 4°C in MilliQ water

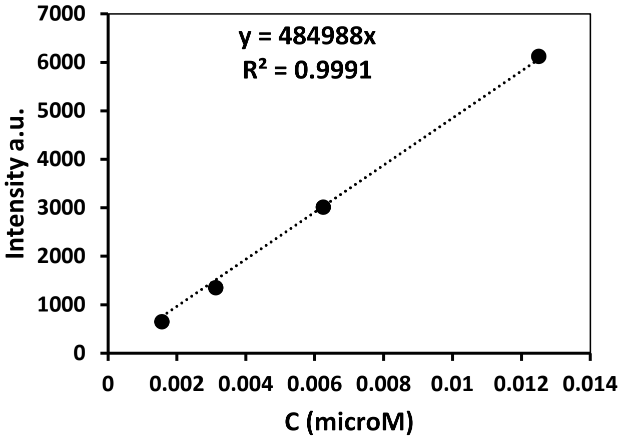

Figure S30. *A representative calibration curve of Cy3-complementary reverse probe for quantification of hybridization density*.
